# Supplementary material for: Telomere DNA length regulation is influenced by seasonal temperature differences in short-lived but not in long-lived reef-building corals
Source: Nat Commun. 2023 Jun 1;14:3038. doi: 10.1038/s41467-023-38499-1 (PMC10235076; doi:10.1038/s41467-023-38499-1)
Supplement: Supplementary file 1 — Supplementary information [file 41467_2023_38499_MOESM1_ESM.pdf]

Supplementary information

Telomere DNA length regulation is influenced by seasonal temperatures differences in short-lived but not in long-lived reef-building corals

Alice Rouan[1,3*], Melanie Pousse[1-3*], Nadir Djerbi[1-3*], Barbara Porro[1-3], Guillaume Bourdin[4], Quentin Carradec[5,6], Benjamin C Hume[7], Julie Poulain[5,6], Julie Lê-Hoang[5,6], Eric Armstrong[5,6], Sylvain Agostini[8], Guillem Salazar[9], Hans-Joachim Ruscheweyh[9], Jean-Marc Aury[5], David A. Paz-García[10], Ryan McMinds[1,11], Marie-Josèphe Giraud-Panis[1-3], Romane Deshuraud[1-3], Alexandre Ottaviani[1-3], Lycia Die Morini[1], Camille Leone[1], Lia Wurzer[1], Jessica Tran[1], Didier Zoccola[12,3], Alexis Pey[1-3], Clémentine Moulin[13], Emilie Boissin[14], Guillaume Iwankow[14], Sarah Romac[14], Colomban de Vargas[15,6], Bernard Banaigs[14], Emmanuel Boss[4], Chris Bowler[16], Eric Douville[17], Michel Flores[18], Stéphanie Reynaud[12,3], Olivier P. Thomas[19], Romain Troublé[13], Rebecca Vega Thurber[20], Serge Planes[14], Denis Allemand[12,3], Stephane Pesant[21], Pierre E. Galand[22], Patrick Wincker[5,6], Shinichi Sunagawa[9], Eric Röttinger[1-3], Paola Furla[1-3], Christian R Voolstra[7], Didier Forcioli[1-3], Fabien Lombard[23] and Eric Gilson[1-3,24]

1 Université Côte d'Azur-CNRS- Inserm - Institute for Research on Cancer and Ageing, Nice (IRCAN), France.
2 Université Côte d'Azur, Institut Fédératif de Recherche - Ressources Marines (IFR MARRES)
3 Laboratoire International Associé Université Côte d'Azur - Centre Scientifique de Monaco (LIA ROPSE), France, Monaco.
4 School of Marine Sciences, University of Maine, Orono, Maine, United States of America
5 Génomique Métabolique, Genoscope, Institut François Jacob, CEA, CNRS, Univ Evry, Université Paris-Saclay, 91057, Evry, France.
6 Research Federation for the Study of Global Ocean Systems Ecology and Evolution, R2022/Tara Oceans GO-SEE, 75016, Paris, France.
7 Department of Biology, University of Konstanz, Konstanz, Germany
8 Shimoda Marine Research Center, University of Tsukuba, Shimoda, Japan.
9 Department of Biology, Institute of Microbiology and Swiss Institute of Bioinformatics, ETH Zurich, 8092 Zurich, Switzerland
10 CONAyT-Centro de Investigaciones Biológicas del Noroeste (CIBNOR), Laboratorio de Necton y Ecología de Arrecifes, La Paz, Baja California Sur 23096, México.
11 University of South Florida Center for Global Health and Infectious Diseases Research, Tampa, FL, United States
12 Centre Scientifique de Monaco, Principality of Monaco
13 Tara Ocean Foundation, 8 rue de Prague, 75012 Paris, France.
14 Laboratoire d'Excellence "CORAIL," PSL Research University: EPHE-UPVD-CNRS, USR 3278 CRIOBE, Université de Perpignan, Perpignan Cedex, France.
15 Sorbonne Université, CNRS, Station Biologique de Roscoff, AD2M, UMR 7144, ECOMAP, Roscoff, France.
16 Institut de Biologie de l'Ecole Normale Supérieure (IBENS), Ecole normale supérieure, CNRS, INSERM, Université PSL, 75005 Paris, France
17 Laboratoire des Sciences du Climat et de l'Environnement, LSCE/IPSL, CEA-CNRS-UVSQ, Université Paris-Saclay, F-91191 Gif-sur-Yvette, France
18 Weizmann Institute of Science, Department of Earth, and Planetary Sciences, 76100 Rehovot, Israel
19 School of Biological and Chemical Sciences, Ryan Institute, University of Galway, University Road H91TK33 Galway, Ireland
20 Oregon State University, Department of Microbiology, 220 Nash Hall, 97331Corvallis OR USA
21 PANGEA, Data Publisher for Earth and Environment Science, Bremen, Germany,
22 Sorbonne Université, CNRS, Laboratoire d'Ecogéochimie des Environnements Benthiques, Observatoire Océanologique de banyuls, Banyuls-sur-Mer, France
23 Sorbonne Université, Institut de la Mer de Villefranche sur mer, Laboratoire d'Océanographie de Villefranche, Villefranche-sur-Mer, France.
24 Department of Medical Genetics, CHU, Nice, France.

* Contributed equally

Eric.Gilson@univ-cotedazur.fr; Alice.Rouan@outlook.fr

This pdf contains Supplementary Figure 1-13 and Supplementary Tables 1-6.

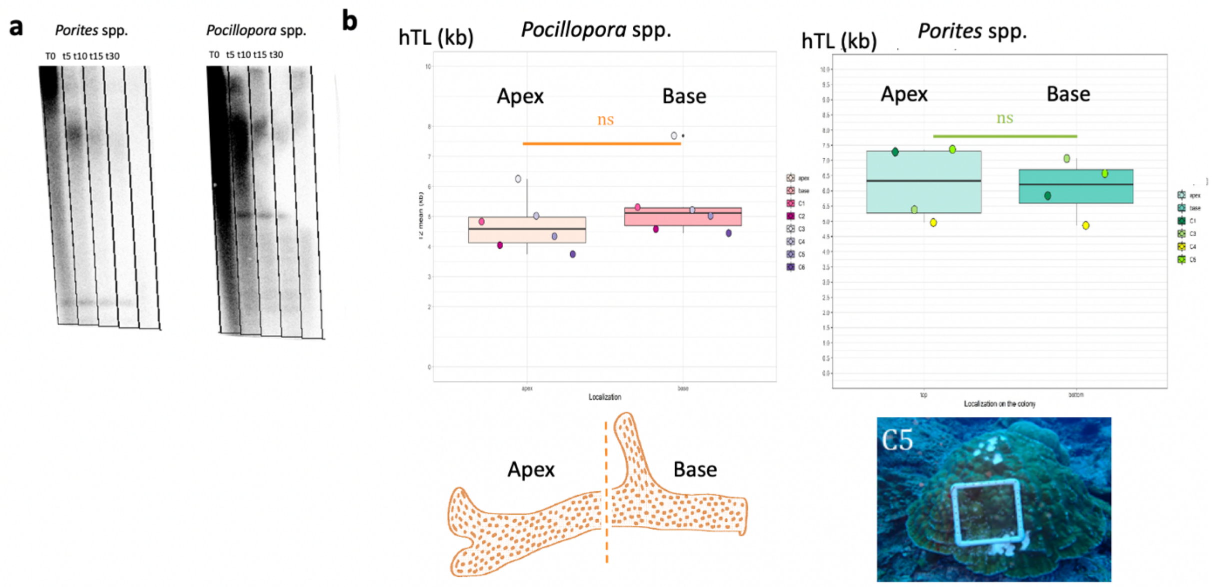

Supplementary Figure 1: Controls of TL measurement in coral.
a) Bal31 Telomere restriction fragment assay. For each lane 5µg of DNA was digested by the exonuclease Bal31 for (t0, 5, 10, 15 and 30 minutes). The reactions were then digested by *Rsa*I and *Hinf*I to perform the Southern blotting TRF analyses. Results show a decrease in the hTL smear with the increase of incubation time with Bal31 while the discrete band (Interstitial Telomeric Sequence) remains, showing that the smear signal is terminally located.
b) Boxplot of mean host TL measures (mean hTL) in 2 different localizations (apex or top of a branch/colony compared to base or edge of a branch/colony) of *Pocillopora* spp. and *Porites* spp. colonies. The colonies were sampled at the Clipperton island. The lower and upper bounds of the box represent the first (Q1) and the third (Q3) quartile, respectively. The entire box represents the interquartile range (IQ). The median is represented as a line across the box. Whiskers extending from Q1 and Q3 are defined as 1.5xIQ. The right box displays the colony part localized at the apex of a branch (*Pocillopora spp.*) or the top of the colony (*Porites spp.*). The right box displays the colony part localized at the base of a branch (*Pocillopora spp.*) or the edge of the colony (*Porites spp.*). Samples are colored by colony (6 colonies for *Pocillopora spp.* and 4 colonies *Porites spp.*) and displayed as dots. Apex and base groups were compared using the Wilcoxon nonparametric test (absence of normal distribution of data) (ns= not significant, pvalue=0.1797 and pvalue=0.4557 respectively). The scheme of the experimental design of branch sectioning is shown below the *Pocilopora* spp hTL results and a picture showing the apex and base sampling of a *Porites* spp colony is found below the *Porites* spp hTL results.
Source data are provided as a Source Data file.

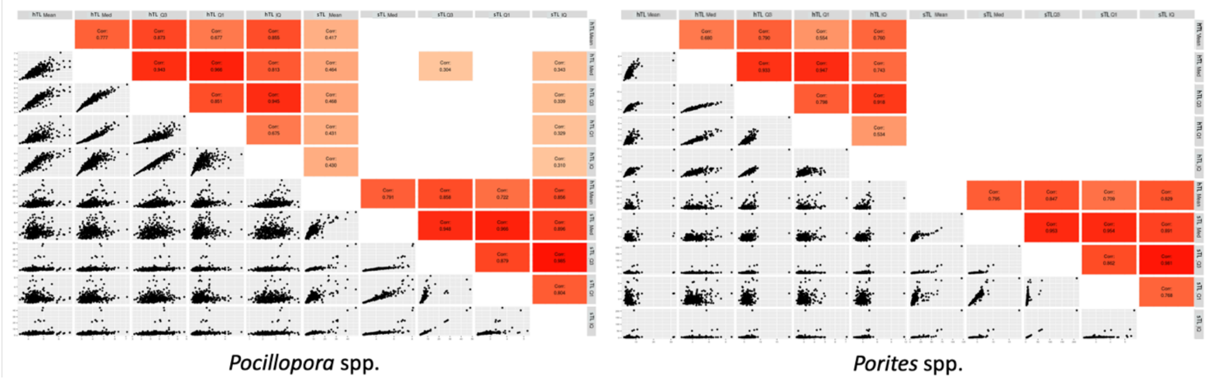

Supplementary Figure 2: Correlation among TL parameters
Correlation matrix between TL (hTL and sTL: mean, median, Q1: 1st quartile, Q3: 3rd quartile, IQ: interquartile distance) in *Pocillopora* and *Porites* samples with the distribution plots in the left half and significant correlation coefficient s (r_s> 0.3, p<0.05) in the right half (n = 363 samples).

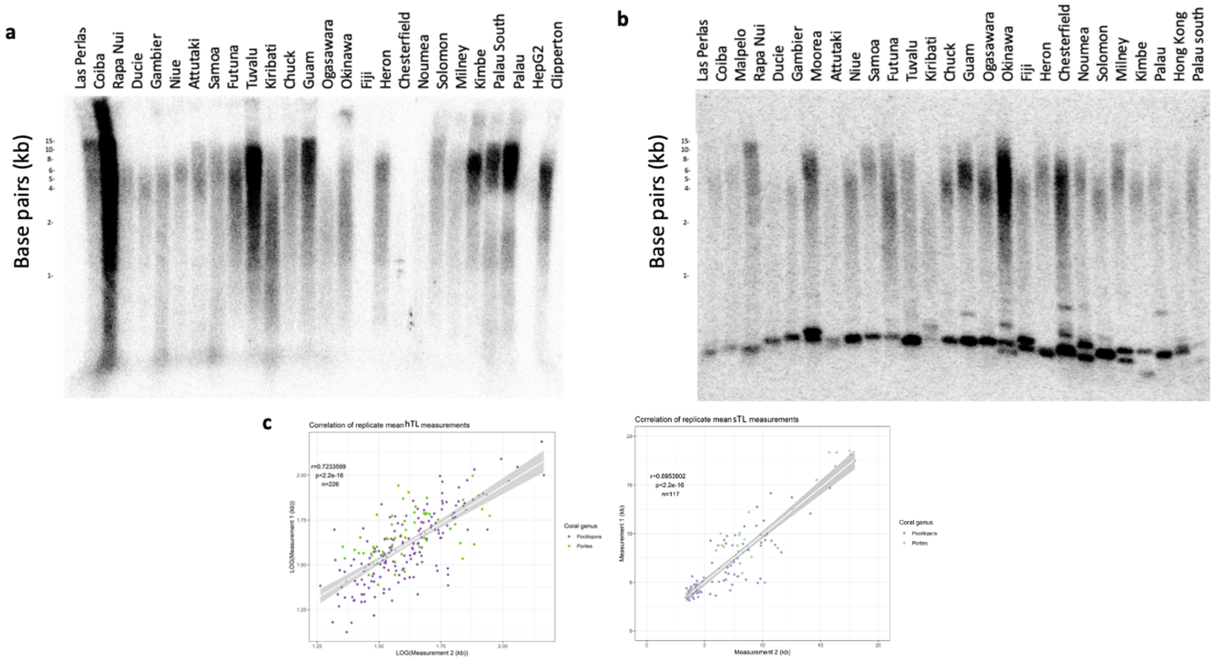

Supplementary Figure 3: Southern control quality

a-b) Examples of Telomere Restriction Fragment (TRF) Southern blotting of *Pocillopora spp.* samples (left) and *Porites spp.* samples (right) hybridized with the radioactive labelled complementary probe to the host telomeric repeat sequence (hTL). The lane names are referring to the sampling island of the samples for *Pocillopora spp.* (blot #150120, samples names are displayed from left to right, M011C06, M021C10, M044C10, M053C10, M063C06, M091C04, M081C01, M102C06, M111C10, M122C01, M131C06, M142C01, M152C10, M163C10, M171C06, M183C07, M193C10, M202C10, M213C01, M223C10, M231C01, M241C01, M252C05, M261C01, HepG2 (human cells), M313C01) and *Porites spp.* (blot #080419, samples names are displayed from left to right, L012C16, L022C13, L031C04, L043C16, L051C16, L061C11, L071C16, L081C11, L093C16, L102C12, L113C11, L123C19, L141C16, L153C11, L163C16, L171C12, L182C16, L191C11, L201C15, L213C11, L222C11, L231C11, L243C11, L263C12, L272C11, L251C13)

c) Correlation analyses between technical replicates of TRF duplicated results for samples loaded at least twice in separate gels. The samples are displayed as dots and colored by genus (*Pocillopora* spp. in purple and *Porites* spp. in green) for mean hTL results on the left and mean sTL on the right. A smoothed linear regression curved is plot as a white line with a grey confidence interval. The hTL (left) data were log transformed, not the sTL (right), to perform a Pearson's correlation test between the technical duplicates (r=0.7233599, pvalue<2.2e^{-16} and r=0.8953802, pvalue<2.2e-^{16}, respectively), n indicates the number of samples.

Source data are provided as a Source Data file, including detailed information of all the blots used in the study to measure TL.

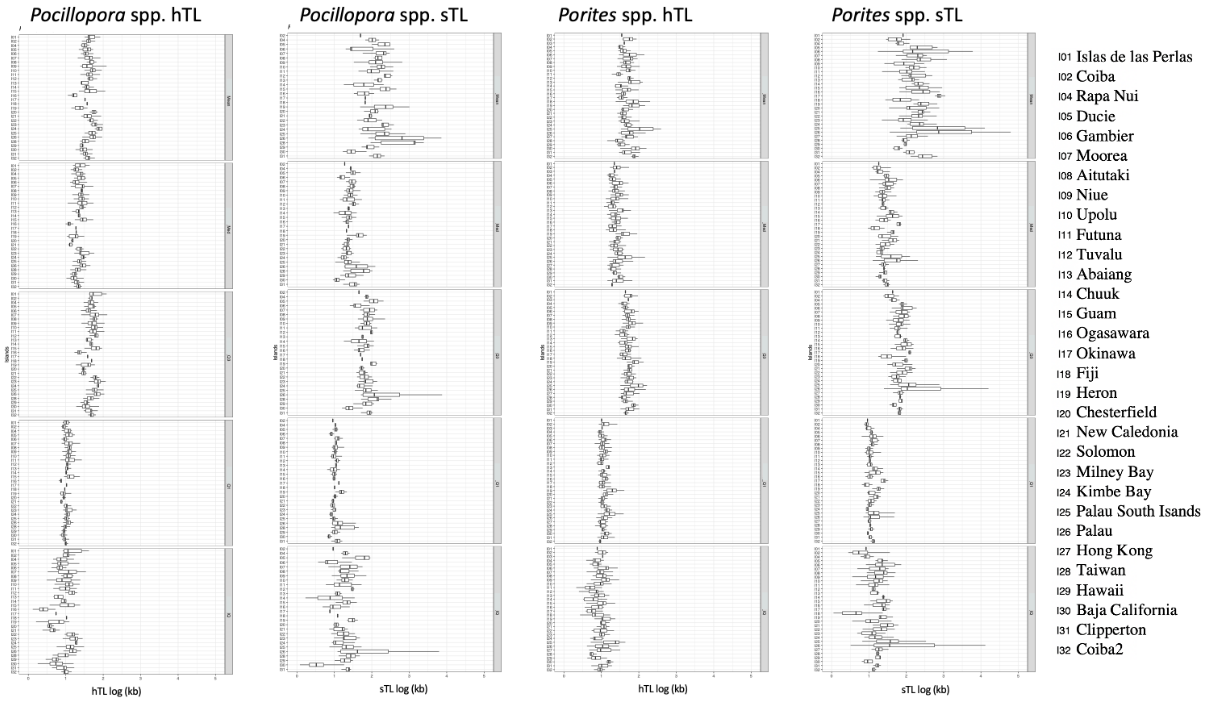

Supplementary Figure 4: Results of TL measurement

Boxplot of coral TLs analyzed in 32 islands of the Pacific Ocean. The various TL parameters analyzed by Southern blotting are shown (n = 444 samples (*Pocillopora* hTL, n = 363 samples (*Pocillopora* sTL), n = 409 samples (*Porites* hTL), n = 341 samples (*Porites* sTL) The boxplots are defined as follows: the lower and upper bounds of the box represent the first (Q1) and the third (Q3) quartile, respectively. The entire box represents the interquartile range (IQ). The median is represented as a line across the box. Whiskers extending from Q1 and Q3 are defined as 1.5xIQ.

Source data are provided as a Source Data file.

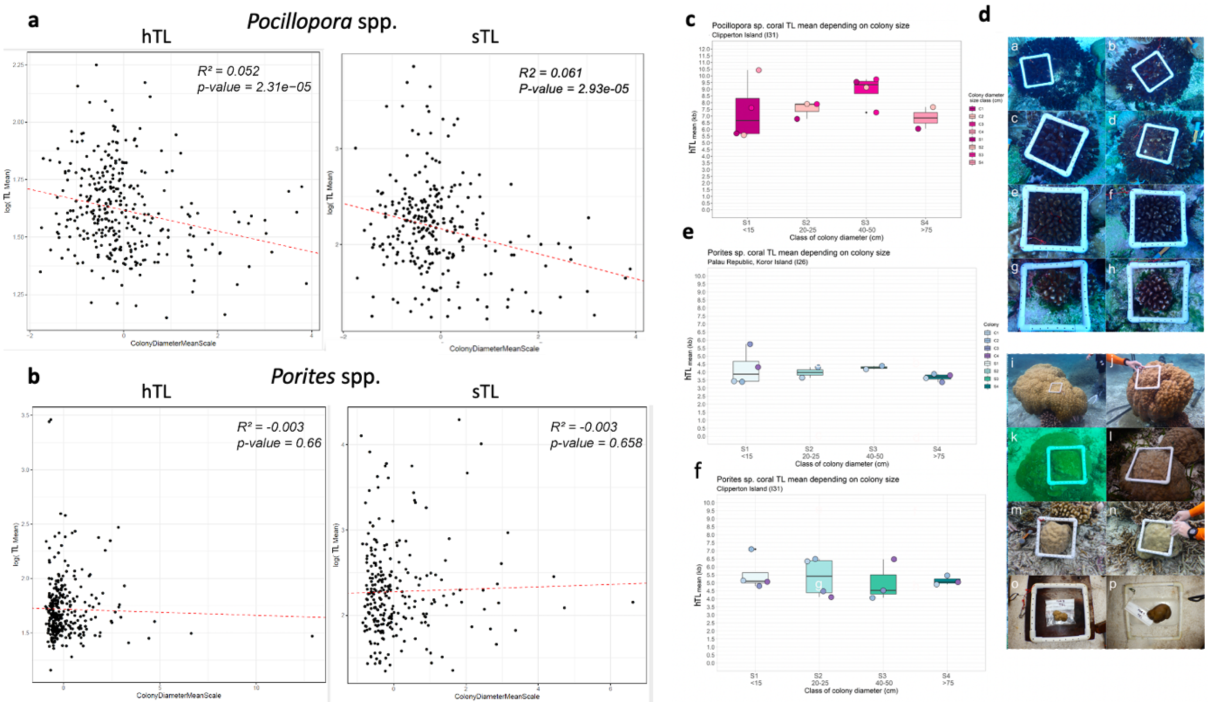

Supplementary Figure 5: TL and colony size

a-b) Log(TL) as a function of Colony diameter mean (scaled: centered and reduced) in *Pocillopora* (a) and *Porites* (b). The red dashed line is extracted from the linear model log(TL) ~ Colony diameter mean. The p-value is obtained using a F-test on the linear model. **c**) Boxplot of mean hTL differences between *Pocillopora* of 4 different size classes from Clipperton Island (Colony diameter: S1< 15 cm ,cm 20 <S2< 25 cm, cm 40 <S3< 50 cm, S4> 75 cm). **d**) Pictures of colonies of different sizes of *Pocillopora* spp. from Clipperton (a-h) and *Porites* spp. from Palau (i-p). Two colonies per size class are displayed ((a-b;i-j) =S4, (c-d;k-l)=S3, (e-f;m-n) =S2, (g-h;o-p) =S1). **e-f**) Boxplot of mean hTL differences between *Porites* samples of 4 different size classes from Palau and Clipperton (Colony diameter: S1< 15 cm, cm 20 <S2< 25 cm, cm 40 <S3< 50 cm, S4> 75 cm). For **c-e-f**) the lower and upper bounds of the box represent the first (Q1) and the third (Q3) quartile, respectively. The entire box represents the interquartile range (IQ). The median is represented as a line across the box. Whiskers extending from Q1 and Q3 are defined as 1.5xIQ. Each individual sample is displayed as a dot on top of the box. TL was not significantly different between size group (Kruskal-Wallis test : (**c**) p = 0.4286, (**e**) p= 0.7748 , (**f**) p= 0.4448).
Source data are provided as a Source Data file.

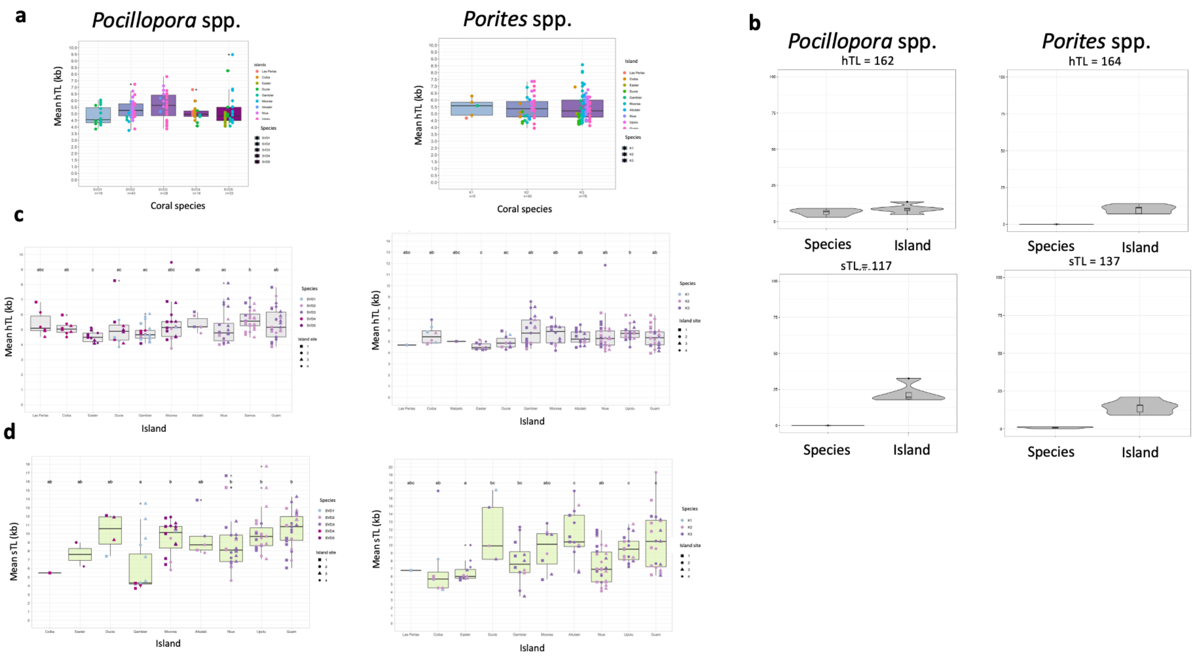

Supplementary Figure 6: Species effect on TL variation
Box plot of mean hTL depending on genetic lineage for 11 islands across the Pacific Ocean. Individuals are colored by island. There are no significant differences neither for *Pocillopora* spp. (left) nor *Porites* spp. (right) (respectively $0.082 < p < 0.476$ and p=0.93). Genetic clusters are equivalent to species and n refers to the number of samples. **b**) Percentage of TL variation explained by the island of origin and host species for 11 islands across the Pacific Ocean. In this analysis, hTL/sTL mean, median, Q1, Q3 and IQ were used. n refers to the number of samples. **c-d**) Box plots of mean hTL and sTL depending on islands of origin for 11 islands for which the host species (genetic cluster) was determined. Individuals are colored by host species. Statistics in **a**, **c** and **d** are calculated using a pairwise wilcoxon test and are displayed as letters for **c** and **d**. Islands sharing at least one letter are not significantly different from each other. For all the boxplots, the lower and upper bounds of the box represent the first (Q1) and the third (Q3) quartile, respectively. The entire box represents the interquartile range (IQ). The median is represented as a line across the box. Whiskers extending from Q1 and Q3 are defined as 1.5xIQ. Each individual sample is displayed as a dot on top of the box.
Source data are provided as a Source Data file.

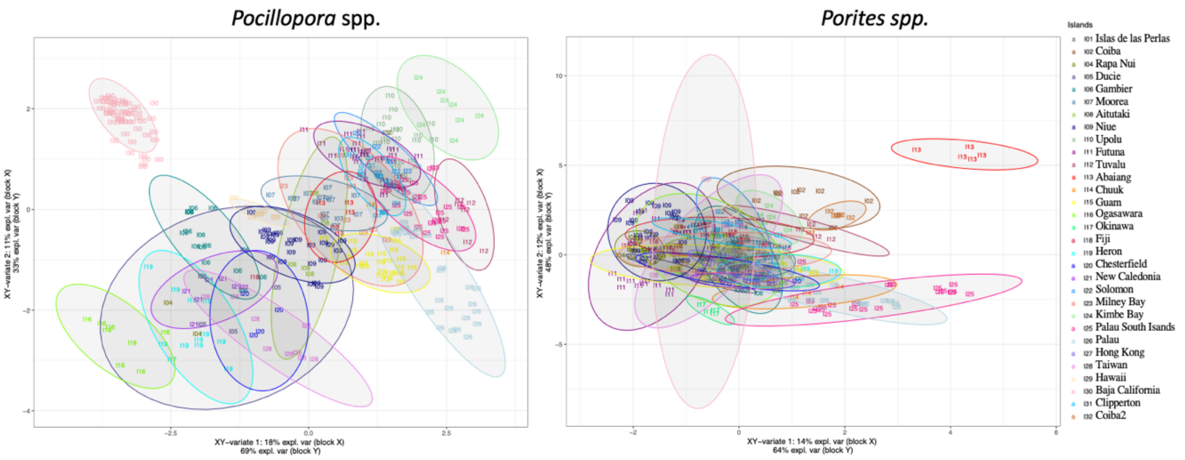

Supplementary Figure 7: sPLS model between TL and environmental variables
Sample plot generated using the *plotIndiv* function corresponding to the sPLS model representing the relationship between hTL and environmental variables. Samples are labelled according to their island of origin.

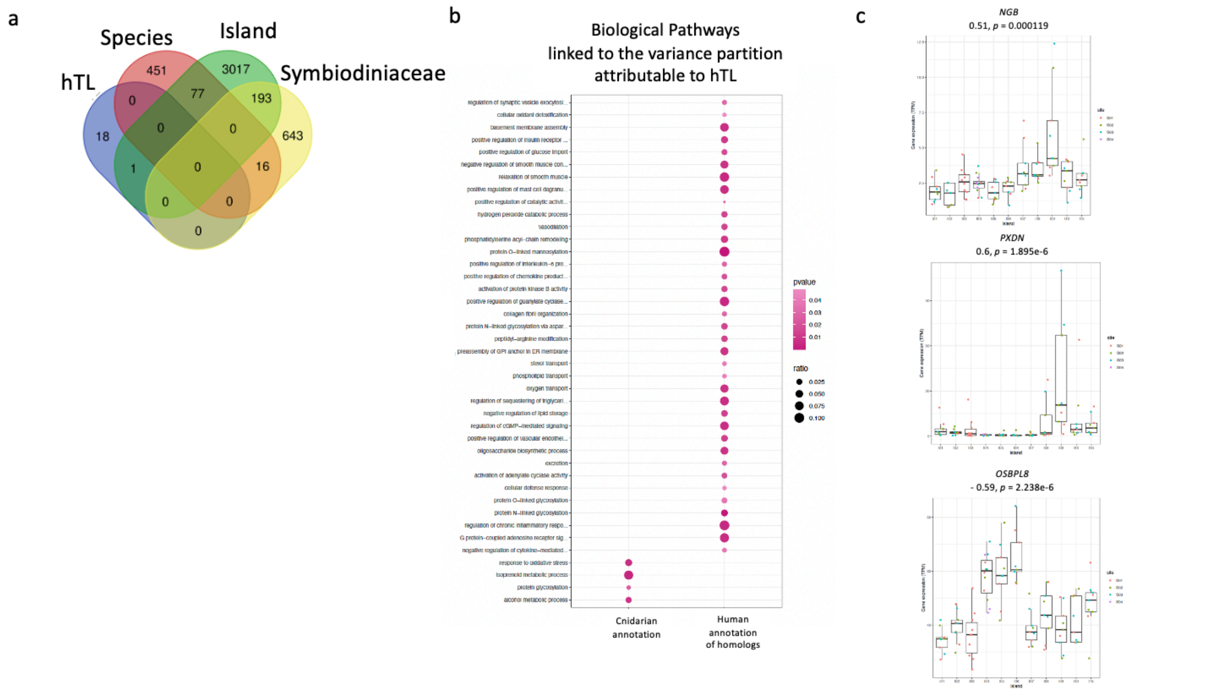

Supplementary Figure 8: Characterization of the expression of hTL-correlated genes
a) Venn diagram of the hTL-correlated genes for which more than 25% of the variation in their expression is explained by one of four predictor variables: the island of origin, the host species (shown as “species”)), the Symbiodiniaceae composition and hTL.
b) Functional enrichment analysis of the genes with expression variations explained by hTL. p-value of the Fisher’s exact test is given as a color gradient.
c) Expression by island of three genes correlated to hTL and involved in redox functions (n = 103 samples). Correlation values from Spearman correlation and adjusted p-value using Benjamini–Hochberg method are given. The boxplots are defined as follows: the lower and upper bounds of the box represent the first (Q1) and the third (Q3) quartile, respectively. The entire box represents the interquartile range (IQ). The median is represented as a line across the box. Whiskers extending from Q1 and Q3 are defined as 1.5xIQ. Source data are provided as a Source Data file.

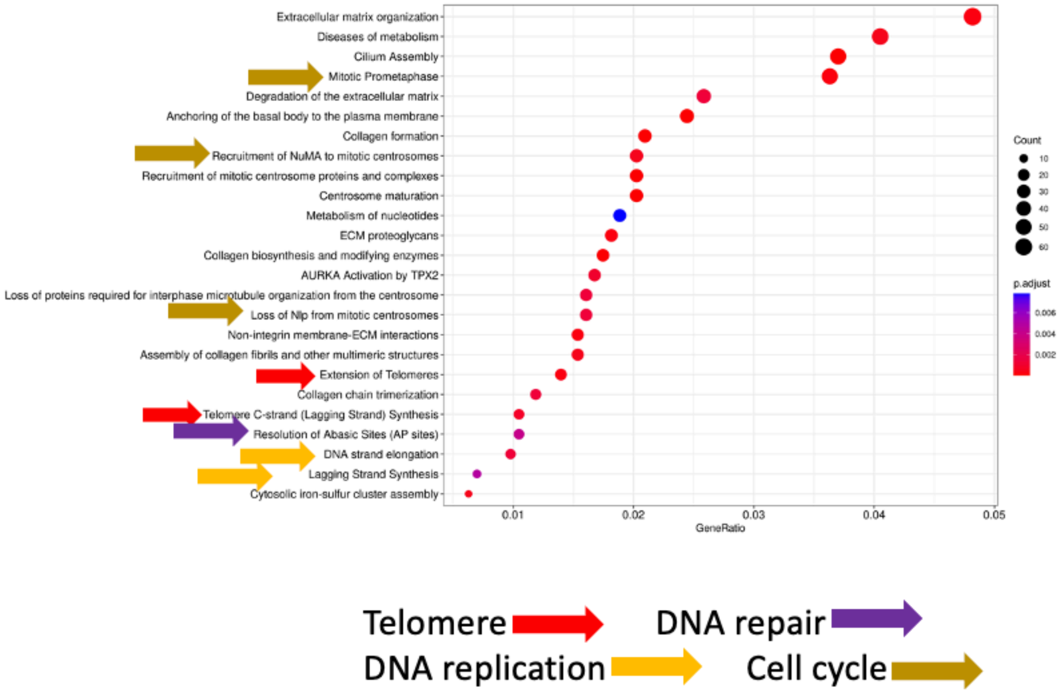

Supplementary Figure 9: Reactome analysis of the expression of hTL-correlated genes

Significant pathways (adjusted p-value < 0.05, hypergeometric test, FDR adjustment) from Reactome analysis of the human homologs of the hTL-correlated genes for which at least 25% of their expression variance is explained by the island of origin (see **Fig. 4a**). Terms and pathways related to telomeres, DNA repair, DNA replication and cell cycle are represented by colored arrows.

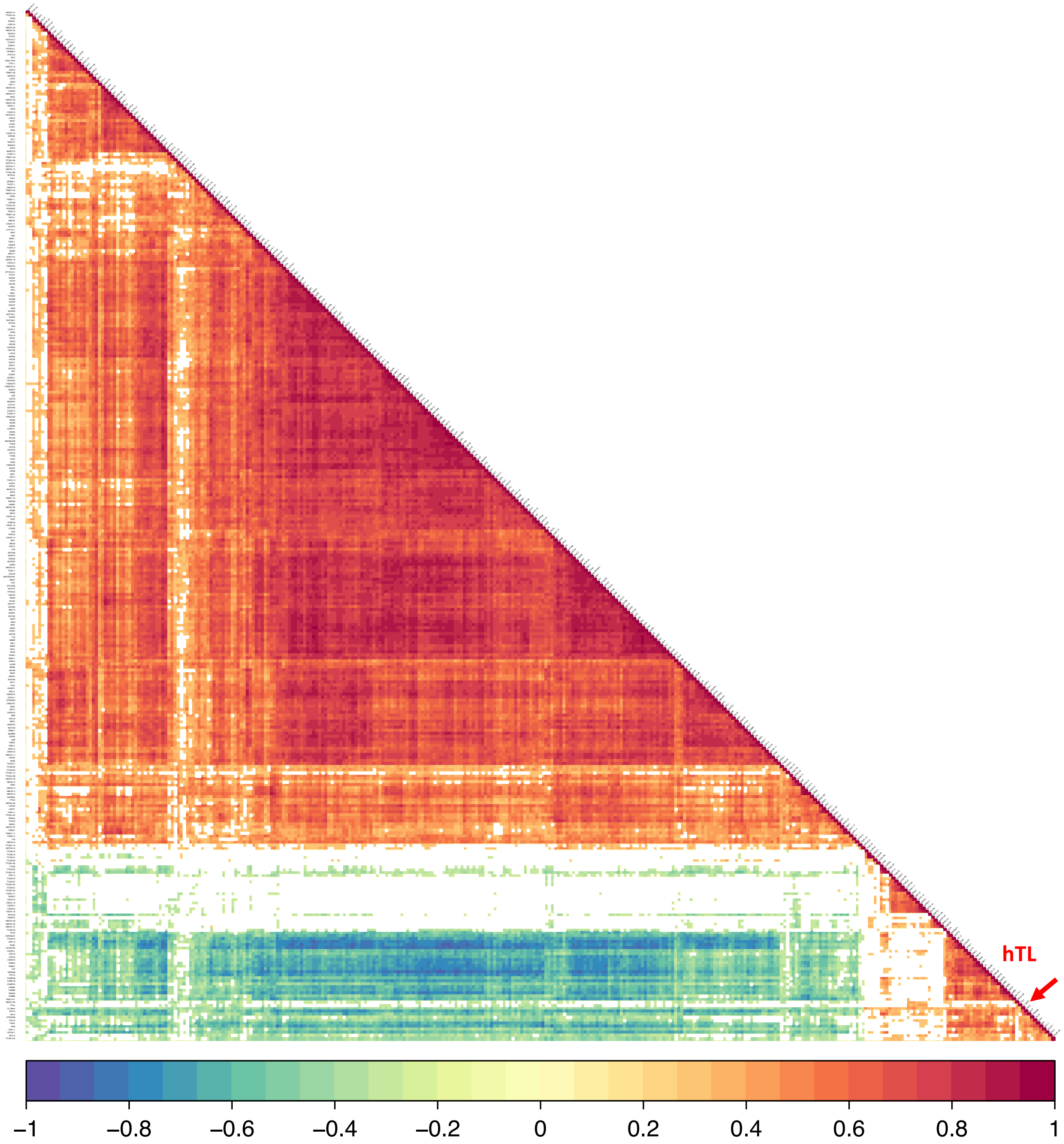

<u>Cell cycle genes</u> whose expression variance

is explained by islands and correlated with hTL

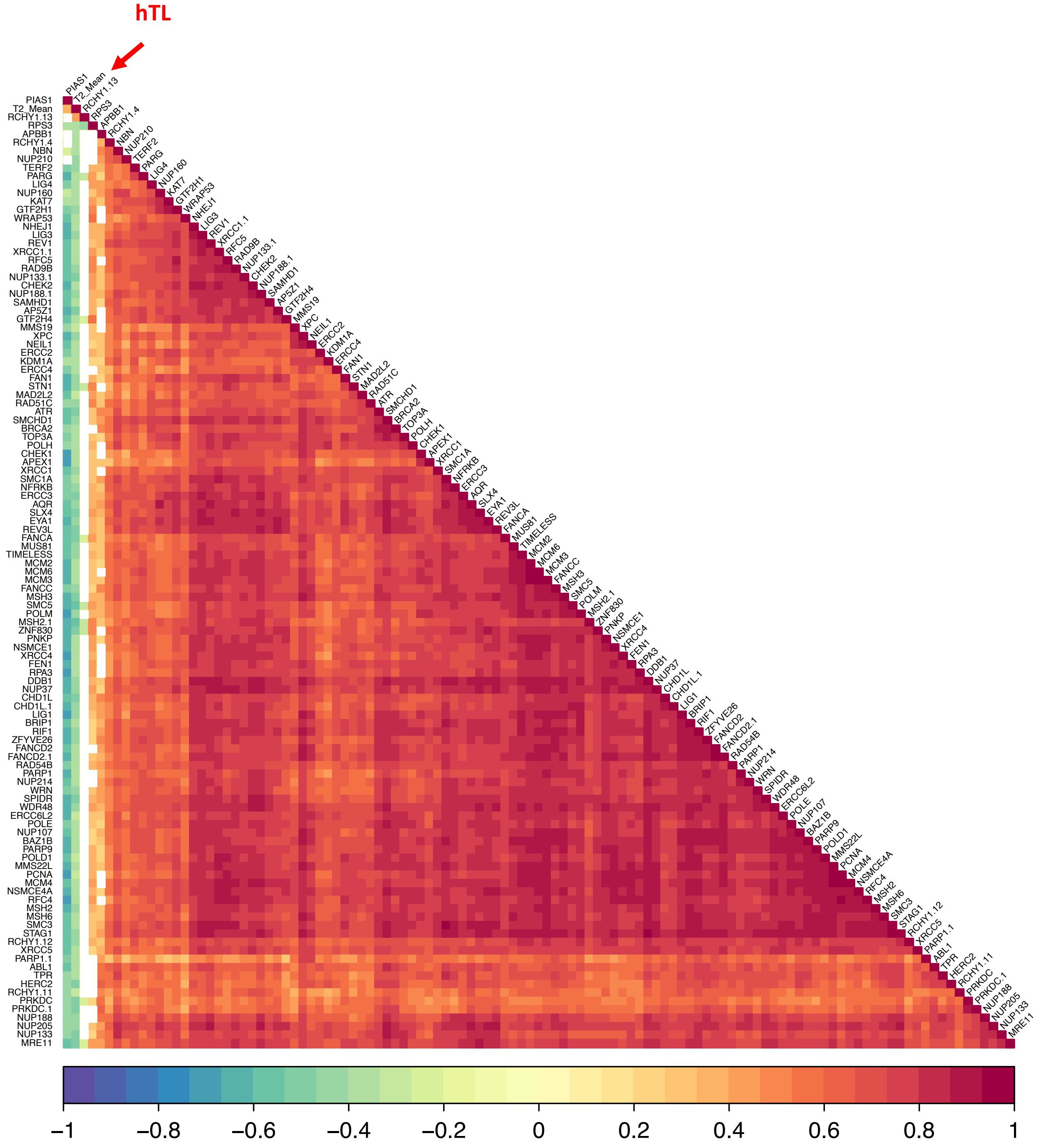

<u>DNA repair genes</u> whose expression variance

is explained by islands and correlated with hTL

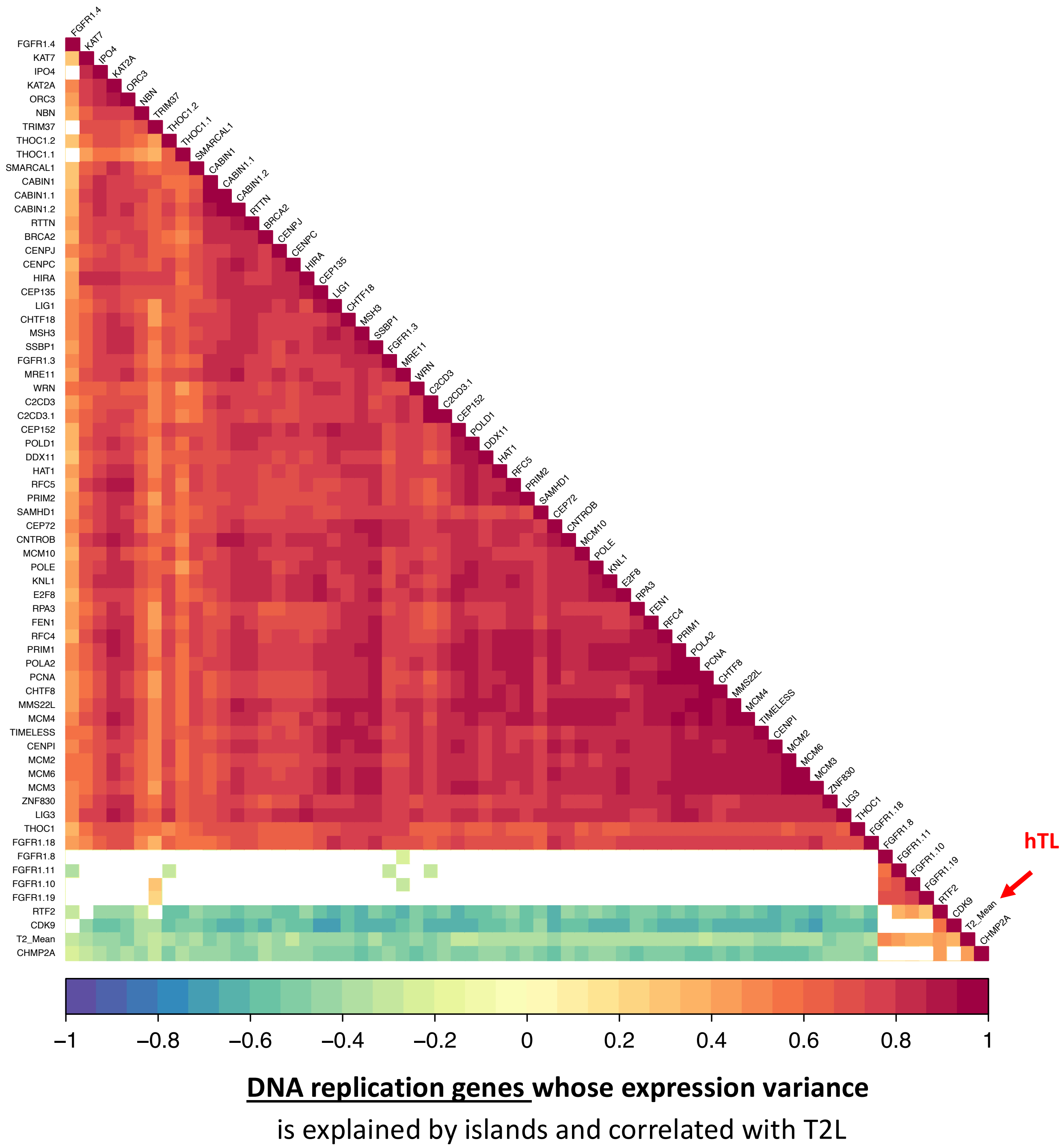

Supplementary Figure 10: Correlation among hTL-correlated genes

Correlograms obtained with Spearman correlation of hTL and genes involved in cell cycle, DNA repair, and DNA replication from the variance partition attributable to island (**Figure 4a-c**). The genes shown here are significantly (r>0.3, p<0.05) correlated with hTL. When the genes are significantly correlated between each other (p<0.05) they are shown according to the color scale of the correlation coefficient. Blank cells indicate genes that are not significantly correlated (p>0.05). *p*-values were adjusted by the Benjamini–Hochberg method.

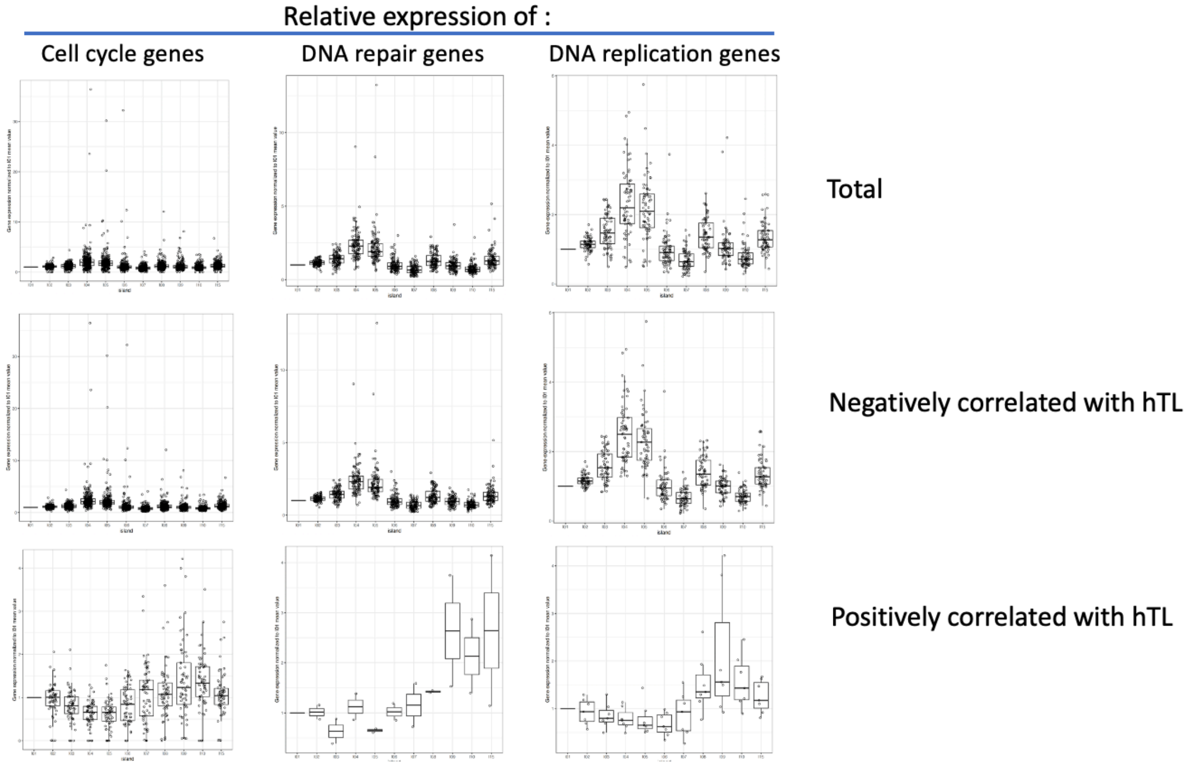

Supplementary Figure 11: Expression of hTL-correlated genes between islands
Gene expression by island for the genes correlated with hTL in the different terms/pathways (Supplementary Fig. 9). The expression of each gene is normalized to the mean expression value of I01 (Las Perlas). The results for total, negatively correlated and positively correlated genes are shown separately. (n = 103 samples). The boxplots are defined as follows: the lower and upper bounds of the box represent the first (Q1) and the third (Q3) quartile, respectively. The entire box represents the interquartile range (IQ). The median is represented as a line across the box. Whiskers extending from Q1 and Q3 are defined as 1.5xIQ. Source data are provided as a Source Data file.

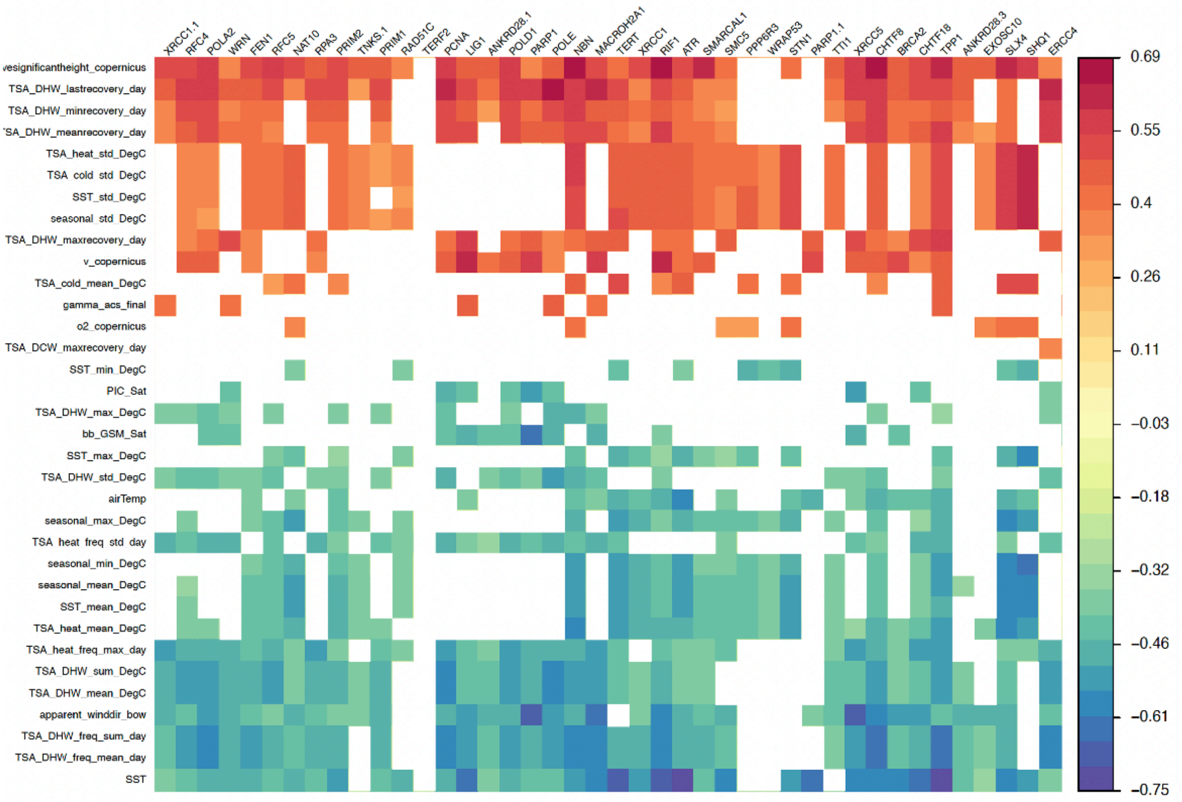

Supplementary Figure 12: Correlation between gene expression and environmental variables

Correlation matrix obtained with Spearman correlation between telomere gene expression and environmental variables in *Pocillopora* spp. (r_s> 0.3, p<0.05) (related to **Figure 5**). *p*-values were adjusted by the Benjamini–Hochberg method.

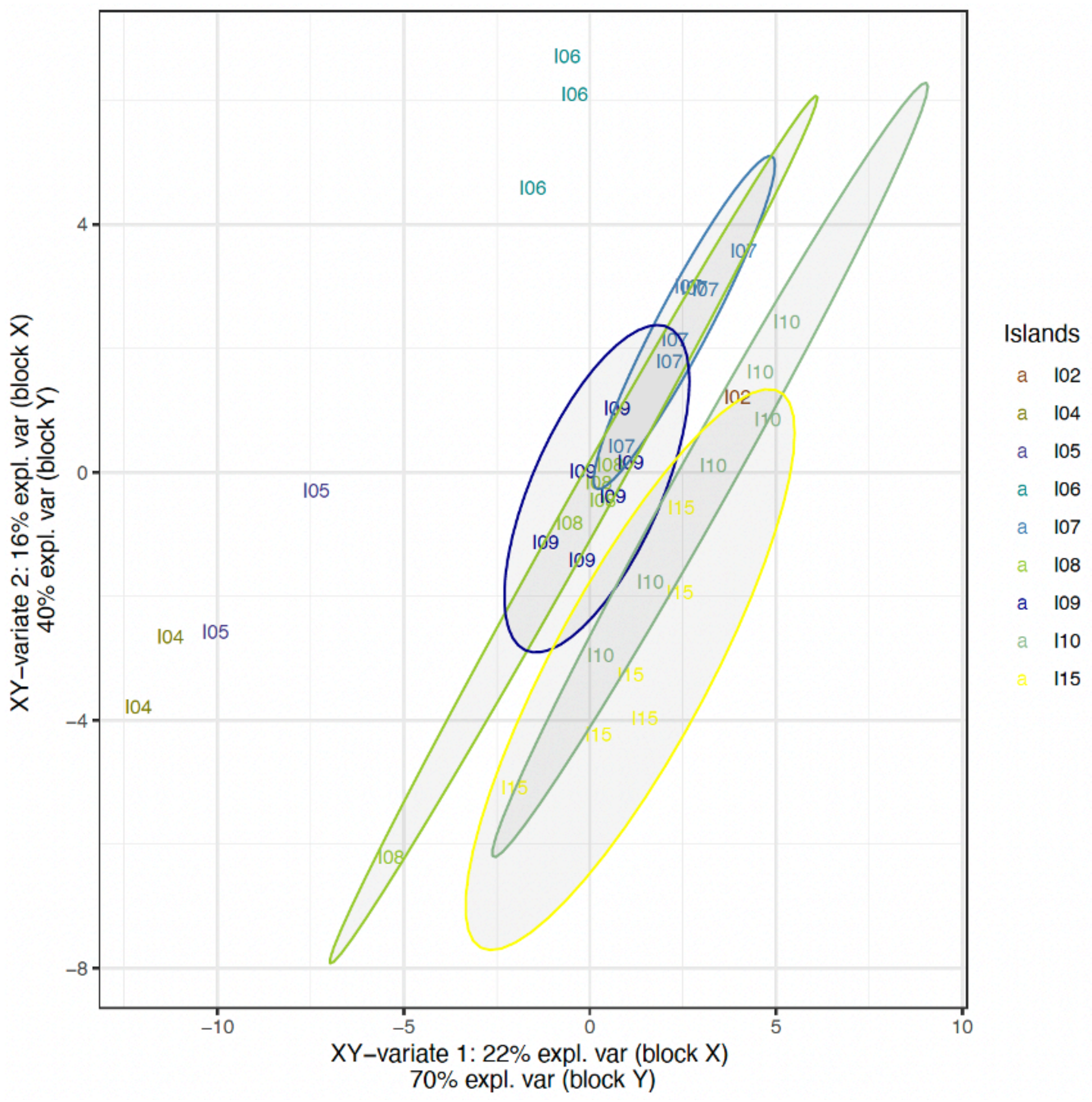

Supplementary Figure 13: sPLS model of gene expression and environmental variables Sample plot generated using the *plotIndiv* function corresponding to the sPLS model representing the relationship between gene expression and environmental variables (related to **Fig. 5**). Samples are labelled according to their island of origin.

Pocillopora hTL													
r.squared	adj.r.squared	sigma	statistic	p.value	df	logLik	AIC	BIC	deviance	df.residual	nobs	error.rate	frml
**0,080219713**	**0,077538138**	**0,178735877**	**29,91514606**	**8,71482E-08**	**1**	**105,5060231**	**-205,0120462**	**-193,4814129**	**10,95765426**	**343**	**345**	**0,03516845**	**log(hTL_Mean) ~ ColonyDiameterMean**
0,081163371	0,075790057	0,178905151	15,10490115	5,17308E-07	2	105,6830921	-203,3661841	-187,9920064	10,94641214	342	345	0,035201757	log(hTL_Mean) ~ poly(ColonyDiameterMean, 2)
0,072889158	0,070186211	0,179446717	26,96655017	3,55605E-07	1	104,1366652	-202,2733304	-190,7426971	11,04498565	343	345	0,035308317	log(hTL_Mean) ~ log(ColonyDiameterMean)
0,069382148	0,066668976	0,960881406	25,57234057	6,95255E-07	1	-474,7639061	955,5278122	967,0584455	316,6895254	343	345	0,189065063	hTL_Mean ~ ColonyDiameterMean
0,070154353	0,064716659	0,961885854	12,90149006	3,96513E-06	2	-474,6207101	957,2414203	972,615598	316,4267437	342	345	0,1892627	hTL_Mean ~ poly(ColonyDiameterMean, 2)
0,062693965	0,059961294	0,964328066	22,9423785	2,48827E-06	1	-475,999199	957,998398	969,5290312	318,9655161	343	345	0,189743235	hTL_Mean ~ log(ColonyDiameterMean)

Pocillopora sTL													
r.squared	adj.r.squared	sigma	statistic	p.value	df	logLik	AIC	BIC	deviance	df.residual	nobs	error.rate	frml
**0,096339772**	**0,093180121**	**0,467604059**	**30,49063585**	**7,53464E-08**	**1**	**-188,732399**	**383,4647979**	**394,4536794**	**62,53491703**	**286**	**288**	**0,049048977**	**log(sTL_Mean) ~ ColonyDiameterMean**
0,096478113	0,090137609	0,468387842	15,21615727	5,26334E-07	2	-188,7103524	385,4207049	400,0725468	62,52534361	285	288	0,049131191	log(sTL_Mean) ~ poly(ColonyDiameterMean, 2)
0,079578651	0,076360395	0,471920703	24,72725601	1,14052E-06	1	-191,3788479	388,7576958	399,7465772	63,69481683	286	288	0,049501768	log(sTL_Mean) ~ log(ColonyDiameterMean)
0,05144397	0,048127341	5,766561821	15,51091865	0,000103123	1	-912,2487062	1830,497412	1841,486294	9510,425278	286	288	0,604879172	sTL_Mean ~ ColonyDiameterMean
0,051449693	0,044793199	5,776652311	7,729248707	0,000538397	2	-912,2478374	1832,495675	1847,147517	9510,367899	285	288	0,605937606	sTL_Mean ~ poly(ColonyDiameterMean, 2)
0,042026741	0,038677184	5,795116231	12,54695553	0,000463229	1	-913,6712829	1833,342566	1844,331447	9604,844428	286	288	0,607874365	sTL_Mean ~ log(ColonyDiameterMean)

Porites hTL													
r.squared	adj.r.squared	sigma	statistic	p.value	df	logLik	AIC	BIC	deviance	df.residual	nobs	error.rate	frml
**0,001333042**	**-0,001837329**	**0,273705377**	**0,420468828**	**0,517175608**	**1**	**-38,06249038**	**82,12498075**	**93,40168607**	**23,59810949**	**315**	**317**	**0,046663208**	**log(hTL_Mean) ~ ColonyDiameterMean**
0,000923327	-0,002248345	0,273761517	0,291116757	0,589886955	1	-38,12750362	82,25500724	93,53171257	23,60779091	315	317	0,046672779	log(hTL_Mean) ~ log(ColonyDiameterMean)
0,00371468	-0,002631086	0,273813784	0,585379334	0,557501137	2	-37,6840454	83,3680908	98,4036979	23,54183231	314	317	0,04668169	log(hTL_Mean) ~ poly(ColonyDiameterMean, 2)
0,002642093	-0,000524123	2,588364947	0,834464046	0,361683519	1	-750,2757101	1506,55142	1517,828126	2110,384426	315	317	0,441282568	hTL_Mean ~ log(ColonyDiameterMean)
0,001304451	-0,001866011	2,590100104	0,411438735	0,521706601	1	-750,4881457	1506,976291	1518,252997	2113,214844	315	317	0,44157839	hTL_Mean ~ ColonyDiameterMean
0,001520221	-0,004839523	2,593940931	0,239038015	0,787528099	2	-750,4538978	1508,907796	1523,943403	2112,75828	314	317	0,442233201	hTL_Mean ~ poly(ColonyDiameterMean, 2)

Porites sTL													
r.squared	adj.r.squared	sigma	statistic	p.value	df	logLik	AIC	BIC	deviance	df.residual	nobs	error.rate	frml
**0,001835688**	**-0,001974099**	**0,52344258**	**0,48183476**	**0,488207434**	**1**	**-202,7013898**	**411,4027795**	**422,1306268**	**71,78593933**	**262**	**264**	**0,045762928**	**log(sTL_Mean) ~ ColonyDiameterMean**
0,00079866	-0,003015086	0,523714421	0,209416056	0,647604739	1	-202,8384581	411,6769161	422,4047635	71,8605203	262	264	0,045786694	log(sTL_Mean) ~ log(ColonyDiameterMean)
0,002722553	-0,00491942	0,52421135	0,356263066	0,700628386	2	-202,5840562	413,1681124	427,4719088	71,72215784	261	264	0,045830139	log(sTL_Mean) ~ poly(ColonyDiameterMean, 2)
0,004252114	0,00045155	9,054554982	1,11881127	0,291148819	1	-955,2587029	1916,517406	1927,245253	21480,06107	262	264	0,791611085	sTL_Mean ~ ColonyDiameterMean
0,00155384	-0,002257023	9,066814683	0,407739688	0,523676759	1	-955,6159122	1917,231824	1927,959672	21538,26766	262	264	0,792682912	sTL_Mean ~ log(ColonyDiameterMean)
0,006598002	-0,001014274	9,061191739	0,866758103	0,421518186	2	-954,9473565	1917,894713	1932,198509	21429,45609	261	264	0,792191316	sTL_Mean ~ poly(ColonyDiameterMean, 2)

Supplementary Table 1 Regression model for the 32 islands

Regression models between TL and colony diameter mean for the 32 islands. r.squared: multiple R^2; adj.r.squared: adjusted R^2; sigma: Residual Standard Error (RSE); statistic: F-test value; p.value: p-value of the F-test; df: degree of freedom of the overall F-test; logLik: log-likelihood of the model; AIC: Akaike's Information Criterion; BIC: Bayesian Information Criterion; df.residual: Residual degrees of freedom; error.rate: RSE divided by the mean of TL; frml: formula of the model

Pocillopora		
Variable	R2_conditior	R2_marginal
hTL_Mean	0.385564468	0.00148136575877723
hTL_Med	0.348170197	0.0180681054639989
hTL_Q3	0.435161114	0.00944006944738771
hTL_Q1	0.297177488	0.0201407626550593
hTL_IQ	0.456166650	0.0044539748181321
sTL_Mean	0.453202394	0.000307014721826544
sTL_Med	0.380238778	1.0723315191928e-07
sTL_Q3	0.268080119	0.00328138754819394
sTL_Q1	0.381634366	0.000340510802483626
sTL_IQ	0.259583308	0.00353045040265365

Porites		
Variable	R2_conditior	R2_marginal
hTL_Mean	0.158962699	0.00711728202982334
hTL_Med	0.200836769	0.000403365007582494
hTL_Q3	0.189367375	0.002667874999667
hTL_Q1	0.152205307	0.001892524897665
hTL_IQ	0.184209367	0.00212670109477538
sTL_Mean	0.234392958	0.00162385206679871
sTL_Med	0.240415948	0.00177927789304019
sTL_Q3	0.087560389	0.00202833862581076
sTL_Q1	0.217739106	0.00774035538845887
sTL_IQ	0.084065245	0.00365071106071572

Supplementary Table 2: R2 values of variance partition
Marginal and conditional R2 related to the variance partition analysis in **Fig. 2**. The marginal R2 considers only the variance of the fixed effects, while the conditional R2 considers both the fixed and random effects (i.e., the total model).

Pocillopora hTL

r.squared	adj.r.squared	sigma	statistic	p.value	df	logLik	AIC	BIC	deviance	df.residual	error.rate	frml
**0,509922374**	**0,427945753**	**0,140663779**	**6,220339018**	**6,28624E-23**	**47**	**200,0697011**	**-304,1394021**	**-122,9609279**	**5,441232123**	**275**	**0,027371958**	**log(hTL_Mean) ~ island+ITS2+16S**
0,509922458	0,425858062	0,140920219	6,065855261	1,58521E-22	48	200,0697286	-302,1394571	-117,1864314	5,441231193	274	0,027421859	log(hTL_Mean) ~ island+ITS2+16S+ColonyDiameterMean
0,485727921	0,410423795	0,142801788	6,45021658	1,69342E-22	42	192,3113262	-298,6226525	-136,316936	5,709858213	280	0,027787996	log(hTL_Mean) ~ island+ITS2
0,485746898	0,408332452	0,143054837	6,27462865	4,33351E-22	43	192,3172672	-296,6345345	-130,5542665	5,709647519	279	0,027837238	log(hTL_Mean) ~ island+ITS2+ColonyDiameterMean
0,427933007	0,371118135	0,147485121	7,532059615	6,37665E-22	30	175,1642397	-288,3284795	-171,3173815	6,351543377	292	0,028699333	log(hTL_Mean) ~ island
0,428013269	0,369045564	0,14772795	7,258435343	1,74523E-21	31	175,1868297	-286,3736595	-165,58801	6,35065225	291	0,028746585	log(hTL_Mean) ~ island+ColonyDiameterMean
0,441554137	0,375396788	0,146982551	6,674302097	4,59153E-21	35	179,044086	-286,088172	-150,2043164	6,200310744	287	0,028601537	log(hTL_Mean) ~ island+16S
0,441604344	0,373269211	0,14723267	6,462332427	1,18953E-20	36	179,0585613	-284,1171226	-144,4587155	6,199753307	286	0,028650208	log(hTL_Mean) ~ island+16S+ColonyDiameterMean
0,271786157	0,225971379	0,163622183	5,932281421	1,04597E-12	20	136,3087187	-230,6174373	-151,3518549	8,085210067	302	0,031839466	log(hTL_Mean) ~ ITS2+16S+ColonyDiameterMean
0,264580127	0,220891818	0,16415819	6,056085297	1,46739E-12	19	134,7233764	-229,4467528	-153,9557219	8,165217152	303	0,031943768	log(hTL_Mean) ~ ITS2+16S
0,224901736	0,189555235	0,167426957	6,362772305	3,21259E-11	15	126,2631125	-220,526225	-160,1334003	8,60575826	307	0,032579842	log(hTL_Mean) ~ ITS2+ColonyDiameterMean
0,209229748	0,175853081	0,168836365	6,268743104	1,76117E-10	14	123,0402694	-216,0805388	-159,4622656	8,779761149	308	0,0328541	log(hTL_Mean) ~ ITS2
0,098721735	0,078629544	0,178517507	4,913438088	2,77014E-05	8	101,9804105	-185,9608209	-151,989857	10,00670913	314	0,034737967	log(hTL_Mean) ~ 16S+ColonyDiameterMean
0,05452907	0,051574474	0,181119533	18,45567327	2,30969E-05	2	94,27348682	-182,5469736	-171,223319	10,49737128	320	0,035244299	log(hTL_Mean) ~ ColonyDiameterMean
0,063854986	0,046023653	0,181648776	3,581055003	0,001899304	7	95,86943923	-175,7388785	-145,5424661	10,39382753	315	0,035347285	log(hTL_Mean) ~ 16S
0,465445932	0,376029615	0,789745787	5,205380271	1,34589E-18	47	-355,4893316	806,9786631	988,1571373	171,5170622	275	0,153677719	hTL_Mean ~ island+ITS2+16S
0,465530181	0,373851051	0,791123266	5,077820688	3,05399E-18	48	-355,463955	808,9279099	993,8809357	171,49003	274	0,153945764	hTL_Mean ~ island+ITS2+16S+ColonyDiameterMean
0,440387896	0,358444696	0,800796892	5,374306735	3,55519E-18	42	-362,8649009	811,7298018	974,0355182	179,5571855	280	0,155828169	hTL_Mean ~ island+ITS2
0,440402659	0,3561622	0,802220146	5,227923259	8,32556E-18	43	-362,8606535	813,721307	979,801575	179,5524486	279	0,156105122	hTL_Mean ~ island+ITS2+ColonyDiameterMean
0,379134608	0,317473319	0,825971658	6,148664979	1,98892E-17	30	-379,5880396	821,1760792	938,1871772	199,2109207	292	0,160726961	hTL_Mean ~ island
0,379371376	0,315389043	0,827231858	5,929314573	4,78414E-17	31	-379,5266303	823,0532606	943,8389101	199,1349513	291	0,160972185	hTL_Mean ~ island+ColonyDiameterMean
0,392946012	0,321030209	0,823816631	5,463973054	1,1705E-16	35	-375,966092	823,9321839	959,8160396	194,7793924	287	0,160307611	hTL_Mean ~ island+16S
0,393151323	0,318886625	0,825116047	5,293919348	2,66944E-16	36	-375,9116312	825,8232624	965,4816696	194,7135163	286	0,160560466	hTL_Mean ~ island+16S+ColonyDiameterMean
0,248233096	0,200936503	0,893708678	5,248435009	6,05248E-11	20	-410,3892731	862,7785462	942,0441287	241,2119905	302	0,173908001	hTL_Mean ~ ITS2+16S+ColonyDiameterMean
0,242718003	0,197730954	0,895499499	5,395286126	6,35372E-11	19	-411,5660858	863,1321715	938,6232024	242,9815637	303	0,174256479	hTL_Mean ~ ITS2+16S
0,201532769	0,165120583	0,913518204	5,534761537	1,62447E-09	15	-420,0923601	872,1847202	932,5775449	256,1962613	307	0,177762764	hTL_Mean ~ ITS2+ColonyDiameterMean
0,188130192	0,15386296	0,91965658	5,490090096	5,7818E-09	14	-422,7723763	875,5447527	932,1630259	260,4966132	308	0,178957239	hTL_Mean ~ ITS2
0,090227021	0,069945458	0,964183151	4,448721252	9,79789E-05	8	-441,1030478	900,2060955	934,1770594	291,9098326	314	0,187621725	hTL_Mean ~ 16S+ColonyDiameterMean
0,047421889	0,044445082	0,977311828	15,93045669	8,15316E-05	2	-448,5053473	903,0106947	914,3343493	305,644291	320	0,190176453	hTL_Mean ~ ColonyDiameterMean
0,062240631	0,044378547	0,977345852	3,484511289	0,002379025	7	-445,9810718	907,9621435	938,1585559	300,8895482	315	0,190183074	hTL_Mean ~ 16S

Pocillopora sTL

r.squared	adj.r.squared	sigma	statistic	p.value	df	logLik	AIC	BIC	deviance	df.residual	error.rate	frml
**0,612088335**	**0,545147826**	**0,315276128**	**9,143765818**	**6,70035E-29**	**40**	**-48,71828634**	**179,4365727**	**326,3599213**	**22,46418239**	**226**	**0,031665361**	**log(sTL_Mean) ~ island+ITS2**
0,613431961	0,544708754	0,315428261	8,926125361	1,44465E-28	41	-48,25680892	180,5136178	331,0204628	22,38637225	225	0,031680641	log(sTL_Mean) ~ island+ITS2+ColonyDiameterMean
0,573388916	0,524991861	0,322185861	11,84759916	9,94728E-31	28	-61,36593993	180,7318799	284,6532728	24,70528744	238	0,032359353	log(sTL_Mean) ~ island
0,575603547	0,525463881	0,322025741	11,48000375	1,96866E-30	29	-60,67370992	181,3474198	288,8523091	24,57703696	237	0,032343271	log(sTL_Mean) ~ island+ColonyDiameterMean
0,61912699	0,54535429	0,315204566	8,392359084	8,84147E-28	44	-46,28283892	182,5656778	343,8230117	22,05656991	222	0,031658173	log(sTL_Mean) ~ island+ITS2+16S
0,620596386	0,545059015	0,315306906	8,215753039	1,77351E-27	45	-45,76873678	183,5374736	348,3783038	21,97147635	221	0,031668452	log(sTL_Mean) ~ island+ITS2+16S+ColonyDiameterMean
0,575826812	0,519632928	0,323998177	10,24714384	7,11035E-29	32	-60,60372335	187,2074467	305,4628249	24,56410756	234	0,032541376	log(sTL_Mean) ~ island+16S
0,578333369	0,520422072	0,323731936	9,986538003	1,23253E-28	33	-59,81545814	187,6309163	309,4697908	24,41895144	233	0,032514636	log(sTL_Mean) ~ island+16S+ColonyDiameterMean
0,384533019	0,339681174	0,379868071	8,573404756	8,7956E-18	19	-110,1115719	260,2231438	331,89307	35,64203851	247	0,038152776	log(sTL_Mean) ~ ITS2+16S
0,386772127	0,339408998	0,379946351	8,166101773	1,76537E-17	20	-109,6268272	261,2536545	336,5070769	35,51237052	246	0,038160638	log(sTL_Mean) ~ ITS2+16S+ColonyDiameterMean
0,329333631	0,291925945	0,393364616	8,803902805	1,51933E-15	15	-121,5350085	275,070017	332,405958	38,83866608	251	0,039508327	log(sTL_Mean) ~ ITS2+ColonyDiameterMean
0,322356799	0,287399015	0,394620062	9,221316687	1,57084E-15	14	-122,9114387	275,8228773	329,575322	39,24269835	252	0,039634421	log(sTL_Mean) ~ ITS2
0,151656079	0,128638996	0,436370126	6,588848749	3,58565E-07	8	-152,7919133	323,5838266	355,8352934	49,12807288	258	0,043827668	log(sTL_Mean) ~ 16S+ColonyDiameterMean
0,129643408	0,109480707	0,441141197	6,429863165	2,47966E-06	7	-156,1989587	328,3979174	357,0658879	50,40283902	259	0,04430686	log(sTL_Mean) ~ 16S
0,064117786	0,060572778	0,453093176	18,08677978	2,9323E-05	2	-165,8529799	337,7059598	348,4564487	54,19746461	264	0,04550728	log(sTL_Mean) ~ ColonyDiameterMean
0,553309808	0,50263487	4,195411127	10,91880588	1,52826E-28	28	-744,0863366	1546,172673	1650,094066	4189,150936	238	0,421374139	sTL_Mean ~ island
0,553419868	0,500659346	4,203734913	10,48927964	4,98811E-28	29	-744,0535626	1548,107125	1655,612015	4188,118771	237	0,422210154	sTL_Mean ~ island+ColonyDiameterMean
0,555366695	0,496462283	4,221364585	9,428269873	1,05063E-26	32	-743,4724934	1552,944987	1671,200365	4169,861036	234	0,423980824	sTL_Mean ~ island+16S
0,555510661	0,494464915	4,229728678	9,099907799	3,16032E-26	33	-743,4294228	1554,858846	1676,69772	4168,510892	233	0,424820888	sTL_Mean ~ island+16S+ColonyDiameterMean
0,575986306	0,502815801	4,194647957	7,871837265	5,1613E-25	40	-737,1571005	1556,314201	1703,23755	3976,486156	226	0,421297489	sTL_Mean ~ island+ITS2
0,576010489	0,500634576	4,203839179	7,641837624	1,45698E-24	41	-737,1495149	1558,29903	1708,805875	3976,259364	225	0,422220626	sTL_Mean ~ island+ITS2+ColonyDiameterMean
0,579993306	0,498640658	4,212223578	7,129372189	1,22955E-23	44	-735,8942519	1561,788504	1723,045838	3938,907699	222	0,42306273	sTL_Mean ~ island+ITS2+16S
0,580024489	0,496409455	4,221586018	6,936844505	3,29691E-23	45	-735,8843771	1563,768754	1728,609584	3938,615261	221	0,424003064	sTL_Mean ~ island+ITS2+16S+ColonyDiameterMean
0,265105274	0,211550193	5,282309997	4,950142321	1,58649E-09	19	-810,3019809	1660,603962	1732,273888	6891,991329	247	0,530538905	sTL_Mean ~ ITS2+16S
0,268928958	0,212464121	5,279247625	4,762768727	2,15102E-09	20	-809,6081706	1661,216341	1736,469764	6856,13205	246	0,53023133	sTL_Mean ~ ITS2+16S+ColonyDiameterMean
0,216584258	0,172887763	5,41027178	4,956559005	3,93141E-08	15	-818,805503	1669,611006	1726,946947	7347,031225	251	0,543390992	sTL_Mean ~ ITS2+ColonyDiameterMean
0,209439288	0,168656394	5,424093152	5,135469015	4,30314E-08	14	-820,0130021	1670,026004	1723,778449	7414,038203	252	0,544779168	sTL_Mean ~ ITS2
0,108100607	0,083901786	5,693874325	4,467184901	0,000103405	8	-836,0542495	1690,108499	1722,359966	8364,412846	258	0,571875156	sTL_Mean ~ 16S+ColonyDiameterMean
0,09418339	0,073199221	5,727037859	4,488306942	0,00024096	7	-838,1135591	1692,227118	1720,895089	8494,931322	259	0,575205999	sTL_Mean ~ 16S
0,033861799	0,030202185	5,858378936	9,252832531	0,002588137	2	-846,6880717	1699,376143	1710,126632	9060,639393	264	0,588397491	sTL_Mean ~ ColonyDiameterMean

Porites hTL

r.squared	adj.r.squared	sigma	statistic	p.value	df	logLik	AIC	BIC	deviance	df.residual	error.rate	frml
**0,353125319**	**0,288437851**	**0,230873973**	**5,458944819**	**1,24687E-14**	**29**	**29,73216286**	**0,535674273**	**112,5359126**	**14,92478161**	**280**	**0,039520909**	**log(hTL_Mean) ~ island**
0,353125534	0,285887686	0,231287317	5,251886334	2,96665E-14	30	29,73221417	2,535571668	118,2691513	14,92477666	279	0,039591665	log(hTL_Mean) ~ island+ColonyDiameterMean
0,361882648	0,280072731	0,232227084	4,423456978	8,41321E-13	36	31,83805651	10,32388698	148,4575142	14,72273131	273	0,039752534	log(hTL_Mean) ~ island+16S
0,36209746	0,277669182	0,232614418	4,288817336	1,73619E-12	37	31,89007517	12,21984967	154,0868182	14,71777515	272	0,039818838	log(hTL_Mean) ~ island+16S+ColonyDiameterMean
0,382966769	0,28554047	0,231343539	3,930835629	4,31768E-12	43	37,02915194	13,94169611	178,2087123	14,23627556	266	0,039601289	log(hTL_Mean) ~ island+ITS2
0,38333612	0,283273679	0,231710244	3,830969104	8,18947E-12	44	37,12166187	15,75667626	183,7570337	14,22775386	265	0,039664062	log(hTL_Mean) ~ island+ITS2+ColonyDiameterMean
0,394080065	0,279446564	0,232328054	3,43773906	8,04573E-11	50	39,83719307	22,32561385	212,726019	13,97986808	259	0,039769818	log(hTL_Mean) ~ island+ITS2+16S
0,395625667	0,278498858	0,232480788	3,377755022	1,19412E-10	51	40,23180058	23,53639883	217,6701452	13,94420775	258	0,039795963	log(hTL_Mean) ~ island+ITS2+16S+ColonyDiameterMean
0,064168139	0,042404608	0,267830222	2,948424938	0,005302565	8	-27,32209783	72,64419566	106,2442672	21,5916414	301	0,045847064	log(hTL_Mean) ~ 16S
0,064470906	0,039523463	0,268232834	2,584269148	0,009636457	9	-27,27210488	74,54420975	111,8776225	21,58465593	300	0,045915983	log(hTL_Mean) ~ 16S+ColonyDiameterMean
0,000630731	-0,00262454	0,274054996	0,193756495	0,660118082	2	-37,47097498	80,94194996	92,14197379	23,05758524	307	0,046912618	log(hTL_Mean) ~ ColonyDiameterMean
0,114017657	0,049189681	0,266879676	1,758772427	0,022584141	22	-18,86495278	83,72990557	169,5967549	20,44150647	287	0,04568435	log(hTL_Mean) ~ ITS2+16S
0,116188516	0,048203017	0,267018111	1,709019101	0,026565971	23	-18,48592801	84,97185603	174,5720467	20,39142012	286	0,045708047	log(hTL_Mean) ~ ITS2+16S+ColonyDiameterMean
0,057934801	0,013074554	0,271900954	1,291450772	0,211333297	15	-28,34777096	88,69554191	148,4290023	21,73545784	294	0,04654389	log(hTL_Mean) ~ ITS2
0,0624623	0,01446549	0,271709283	1,301384392	0,199898721	16	-27,60346489	89,20692978	152,6737315	21,63099876	293	0,04651108	log(hTL_Mean) ~ ITS2+ColonyDiameterMean
0,243995634	0,168395197	2,378963139	3,227436829	3,54171E-07	29	-691,0251769	1442,050354	1554,050592	1584,650373	280	0,407229904	hTL_Mean ~ island
0,245027523	0,166553681	2,381595685	3,12241019	5,70051E-07	30	-690,8141519	1443,628304	1559,361883	1582,487444	279	0,407680542	hTL_Mean ~ island+ColonyDiameterMean
0,255280466	0,159803602	2,391220504	2,673741643	4,57898E-06	36	-688,7015791	1451,403158	1589,536785	1560,996392	273	0,409328114	hTL_Mean ~ island+16S
0,256968507	0,158626103	2,392895514	2,612998033	6,28005E-06	37	-688,3509794	1452,701959	1594,568927	1557,458111	272	0,409614842	hTL_Mean ~ island+16S+ColonyDiameterMean
0,266242338	0,150385865	2,404584719	2,298035938	3,86814E-05	43	-686,4105185	1460,821037	1625,088053	1538,019361	266	0,411615795	hTL_Mean ~ island+ITS2
0,26866385	0,149994211	2,405138887	2,263964495	4,63628E-05	44	-685,8998018	1461,799604	1629,799961	1532,943663	265	0,411710657	hTL_Mean ~ island+ITS2+ColonyDiameterMean
0,277381418	0,140669794	2,418294885	2,028952699	0,000224105	50	-684,047089	1470,094178	1660,494583	1514,670889	259	0,413962695	hTL_Mean ~ island+ITS2+16S
0,281174676	0,141867443	2,416609109	2,01837815	0,000226823	51	-683,2339325	1470,467865	1664,601611	1506,719893	258	0,413674125	hTL_Mean ~ island+ITS2+16S+ColonyDiameterMean
0,050020722	0,02792818	2,572044007	2,264145209	0,029314181	8	-726,3118985	1470,623797	1504,223869	1991,238523	301	0,440281405	hTL_Mean ~ 16S
0,050785104	0,025472707	2,575290476	2,006333261	0,045467007	9	-726,187533	1472,375066	1509,708479	1989,636311	300	0,440837134	hTL_Mean ~ 16S+ColonyDiameterMean
0,000891417	-0,00236301	2,611810923	0,273909313	0,601098901	2	-734,1022971	1474,204594	1485,404618	2094,217783	307	0,447088689	hTL_Mean ~ ColonyDiameterMean
0,05500712	0,010007459	2,595644312	1,222389649	0,257766377	15	-725,4987992	1482,997598	1542,731059	1980,786602	294	0,444321295	hTL_Mean ~ ITS2
0,059764098	0,011629154	2,593517494	1,241594836	0,239619855	16	-724,7191011	1483,438202	1546,905004	1970,815566	293	0,443957227	hTL_Mean ~ ITS2+ColonyDiameterMean
0,091053857	0,024545603	2,57651517	1,369061001	0,132019011	22	-719,4900548	1484,98011	1570,846959	1905,229531	287	0,441046777	hTL_Mean ~ ITS2+16S
0,093864728	0,024162014	2,577021716	1,346643814	0,140321885	23	-719,011531	1486,023062	1575,623253	1899,337704	286	0,441133487	hTL_Mean ~ ITS2+16S+ColonyDiameterMean

Porites sTL

r.squared	adj.r.squared	sigma	statistic	p.value	df	logLik	AIC	BIC	deviance	df.residual	error.rate	frml
**0,437316149**	**0,334071405**	**0,426855065**	**4,235723141**	**2,52681E-12**	**41**	**-124,6984727**	**333,3969455**	**482,7837241**	**39,72074379**	**218**	**0,036971595**	**log(sTL_Mean) ~ island+ITS2**
0,437400244	0,331102595	0,427805499	4,114862797	5,21839E-12	42	-124,679117	335,3582339	488,3018406	39,71480735	217	0,037053916	log(sTL_Mean) ~ island+ITS2+ColonyDiameterMean
0,372365697	0,299005844	0,437949269	5,075878419	1,63018E-12	28	-138,8450202	335,6900404	438,8380542	44,30569895	231	0,037932508	log(sTL_Mean) ~ island
0,404456706	0,314061742	0,433220621	4,474327856	3,71403E-12	35	-132,0483955	336,0967909	464,1426011	42,04034383	224	0,037522941	log(sTL_Mean) ~ island+16S
0,460428162	0,340239174	0,424873721	3,830868123	1,21413E-11	48	-119,2669845	336,5339689	510,818544	38,0892302	211	0,036799983	log(sTL_Mean) ~ island+ITS2+16S
0,372991768	0,296660331	0,438681342	4,886476433	3,41209E-12	29	-138,7157784	337,4315568	444,1363987	44,26150365	230	0,037995915	log(sTL_Mean) ~ island+ColonyDiameterMean
0,405055001	0,311677983	0,433972728	4,33784469	7,32005E-12	36	-131,9182318	337,8364637	469,4391019	41,99810928	223	0,037588084	log(sTL_Mean) ~ island+16S+ColonyDiameterMean
0,460453367	0,337128423	0,425874176	3,733659633	2,40378E-11	49	-119,2609348	338,5218696	516,3632727	38,08745089	210	0,036886636	log(sTL_Mean) ~ island+ITS2+16S+ColonyDiameterMean
0,227644199	0,159207609	0,47963514	3,326352164	3,5866E-06	22	-165,7146708	377,4293417	459,2363871	54,52181861	237	0,041543085	log(sTL_Mean) ~ ITS2+16S
0,227961703	0,155992031	0,480551438	3,16746898	6,52544E-06	23	-165,6614244	379,3228488	464,6867223	54,49940552	236	0,041622449	log(sTL_Mean) ~ ITS2+16S+ColonyDiameterMean
0,164160274	0,116202257	0,491748509	3,422999624	4,38293E-05	15	-175,944077	383,8881539	440,7974029	59,00324936	244	0,042592271	log(sTL_Mean) ~ ITS2
0,165401424	0,113882994	0,492393309	3,210529175	7,42073E-05	16	-175,7516377	385,5032754	445,9693524	58,91563461	243	0,04264812	log(sTL_Mean) ~ ITS2+ColonyDiameterMean
0,108904985	0,08405373	0,500612405	4,382272982	0,00013158	8	-184,2339134	386,4678269	418,4792794	62,9038077	251	0,043360008	log(sTL_Mean) ~ 16S
0,109781558	0,081294568	0,501365851	3,85374368	0,000267917	9	-184,1064612	388,2129223	423,781203	62,84192901	250	0,043425267	log(sTL_Mean) ~ 16S+ColonyDiameterMean
0,00076489	-0,00312318	0,523894382	0,196727177	0,657748814	2	-199,0667186	404,1334372	414,8039214	70,5375882	257	0,045376552	log(sTL_Mean) ~ ColonyDiameterMean
0,330596405	0,252354426	7,877214002	4,225307318	6,56066E-10	28	-887,2582803	1832,516561	1935,664574	14333,6656	231	0,682276467	sTL_Mean ~ island
0,330983924	0,249538489	7,892034457	4,06387323	1,30928E-09	29	-887,1832907	1834,366581	1941,071423	14325,36781	230	0,683560125	sTL_Mean ~ island+ColonyDiameterMean
0,378926323	0,26496785	7,810483667	3,325126371	8,46004E-09	41	-877,5538815	1839,107763	1988,494542	13298,79682	218	0,67649669	sTL_Mean ~ island+ITS2
0,348357373	0,249447331	7,89251376	3,521961646	8,7392E-09	35	-883,7759132	1839,551826	1967,597637	13953,35725	224	0,683601639	sTL_Mean ~ island+16S
0,378948351	0,261606795	7,82832066	3,229447132	1,56819E-08	42	-877,5492885	1841,098577	1994,042184	13298,32514	217	0,678041622	sTL_Mean ~ island+ITS2+ColonyDiameterMean
0,348738237	0,246522265	7,907878223	3,411778322	1,59657E-08	36	-883,7002026	1841,400405	1973,003043	13945,20197	223	0,684932416	sTL_Mean ~ island+16S+ColonyDiameterMean
0,388551301	0,252351828	7,877227691	2,852810601	1,60172E-07	48	-875,5312652	1849,06253	2023,347105	13092,7011	211	0,682277653	sTL_Mean ~ island+ITS2+16S
0,388553244	0,248793985	7,895948177	2,780161026	2,75748E-07	49	-875,5308537	1851,061707	2028,903111	13092,6595	210	0,683899107	sTL_Mean ~ island+ITS2+16S+ColonyDiameterMean
0,14918246	0,10036506	8,640878887	3,055928024	0,000223239	15	-918,3137452	1868,62749	1925,536739	18218,20826	244	0,748420484	sTL_Mean ~ ITS2
0,190312077	0,118567577	8,553015751	2,652636481	0,000191795	22	-911,8971749	1869,79435	1951,601395	17337,51659	237	0,740810313	sTL_Mean ~ ITS2+16S
0,149193393	0,096674467	8,658584588	2,840754818	0,000409707	16	-918,3120811	1870,624162	1931,090239	18217,97416	243	0,749954045	sTL_Mean ~ ITS2+ColonyDiameterMean
0,190346719	0,114870566	8,570934031	2,521945161	0,000323896	23	-911,8916341	1871,783268	1957,147142	17336,7748	236	0,742362285	sTL_Mean ~ ITS2+16S+ColonyDiameterMean
0,074649912	0,048843336	8,884864081	2,892670141	0,006339377	8	-929,1884352	1876,37687	1908,388323	19814,14325	251	0,769553001	sTL_Mean ~ 16S
0,077488234	0,047967857	8,888952116	2,624906684	0,00891321	9	-928,7906101	1877,58122	1913,149501	19753,36743	250	0,769907082	sTL_Mean ~ 16S+ColonyDiameterMean
0,003111689	-0,00076725	9,113628137	0,802200299	0,371274449	2	-938,8318598	1883,66372	1894,334204	21345,96198	257	0,789367155	sTL_Mean ~ ColonyDiameterMean

Supplementary Table 3: Regression models

Regression models between TL, island of sampling, ITS2 clusters, 16S clusters and colony diameter mean for the 32 islands. r.squared: multiple R2; adj.r.squared: adjusted R2; sigma: Residual Standard Error (RSE); statistic: F-test value; p.value: p-value of the F-test; df: degree of freedom of the overall F-test; logLik: log-likelihood of the model; AIC: Akaike's Information Criterion; BIC: Bayesian Information Criterion; df.residual: Residual degrees of freedom; error.rate: RSE divided by the mean of TL; frml: formula of the model.

Pocillopora		
Variable	R2_conditior	R2_marginal
hTL_Mean	0.116721607	0
hTL_Med	0.154398860	0
hTL_Q3	0.164377142	0
hTL_Q1	0.140371332	0
hTL_IQ	0.186773575	0
sTL_Mean	0.196642379	0
sTL_Med	0.178153814	0
sTL_Q3	0.228716366	0
sTL_Q1	0.184217637	0
sTL_IQ	0.326476388	0
Porites		
Variable	R2_conditior	R2_marginal
hTL_Mean	0.069784033	0
hTL_Med	0.115918231	0
hTL_Q3	0.140117746	0
hTL_Q1	0.073030285	0
hTL_IQ	0.111594215	0
sTL_Mean	0.158229362	0
sTL_Med	0.225056949	0
sTL_Q3	0.121876166	0
sTL_Q1	0.172565639	0
sTL_IQ	0.096695425	0

Supplementary Table 4: R2 of variance partition analysis

Marginal and conditional R2 related to the variance partition analysis in **Supplementary Fig. 6b**. The marginal R2 considers only the variance of the fixed effects, while the conditional R2 considers both the fixed and random effects (i.e., the total model). Since the island and the host species are random effects, the marginal R2 is null.

Pocillopora hTL

r.squared	adj.r.squared	sigma	statistic	p.value	df	logLik	AIC	BIC	deviance	df.residual	error.rate	frml
**0,202067683**	**0,13197903**	**0,158884393**	**2,883030063**	**0,000969446**	**14**	**75,46477246**	**-120,9295449**	**-74,61559989**	**3,736149036**	**148**	**0,030391336**	**log(hTL_Mean) ~ island+genetic_clusters**
0,140855452	0,08998505	0,162682334	2,768907844	0,004984471	10	69,47778666	-116,9555733	-82,99201363	4,022762341	152	0,031117805	log(hTL_Mean) ~ island
0,048999584	0,02477027	0,168410667	2,022326864	0,093888148	5	61,25002398	-110,500048	-91,97446995	4,452857982	157	0,032213518	log(hTL_Mean) ~ genetic_clusters
0,186960057	0,11554439	0,895032951	2,617913614	0,0026232	14	-204,5819848	439,1639696	485,4779146	118,5604296	148	0,171201506	hTL_Mean ~ island+genetic_clusters
0,123802299	0,07192217	0,91683926	2,386314484	0,014838497	10	-210,6417173	443,2834347	477,2469944	127,7703226	152	0,175372607	hTL_Mean ~ island
0,050992184	0,02681364	0,93885605	2,108984969	0,082205165	5	-217,1075652	446,2151305	464,7407085	138,3877573	157	0,179583969	hTL_Mean ~ genetic_clusters

Pocillopora sTL

r.squared	adj.r.squared	sigma	statistic	p.value	df	logLik	AIC	BIC	deviance	df.residual	error.rate	frml
**0,42082145**	**0,35399316**	**0,27223361**	**6,297055076**	**2,93173E-08**	**13**	**-6,897418548**	**41,7948371**	**80,46527218**	**7,707558409**	**104**	**0,029625902**	**log(sTL_Mean) ~ island+genetic_clusters**
0,363789458	0,31666275	0,279988846	7,719390594	3,8483E-08	9	-12,39166501	44,78333003	72,40506938	8,466525415	108	0,030469868	log(sTL_Mean) ~ island
0,193020434	0,16419974	0,309652389	6,697285016	7,18655E-05	5	-26,30113962	64,60227925	81,17532286	10,73907543	112	0,033698012	log(sTL_Mean) ~ genetic_clusters
0,254461129	0,19923603	2,562716831	4,60770776	7,3698E-05	9	-271,438261	562,8765221	590,4982614	709,2918959	108	0,278888407	sTL_Mean ~ island
0,296376284	0,21518893	2,53706105	3,650522866	0,000140044	13	-268,0532431	564,1064861	602,7769212	669,4145922	104	0,276096409	sTL_Mean ~ island+genetic_clusters
0,133314859	0,10236182	2,713307512	4,30700363	0,002804756	5	-280,246517	572,493034	589,0660776	824,5482171	112	0,295276481	sTL_Mean ~ genetic_clusters

Porites hTL

r.squared	adj.r.squared	sigma	statistic	p.value	df	logLik	AIC	BIC	deviance	df.residual	error.rate	frml
**0,142073195**	**0,08599955**	**0,166593368**	**2,533689203**	**0,00747818**	**11**	**66,90791282**	**-109,8158256**	**-72,61742851**	**4,246262588**	**153**	**0,030325808**	**log(T2_Mean) ~ island**
0,14635611	0,07237364	0,16783056	1,978253888	0,026132454	14	67,31829582	-104,6365916	-58,13859523	4,225064531	150	0,03055102	log(T2_Mean) ~ island+genetic_clusters
0,002596979	-0,01610433	0,175652224	0,13886619	0,936634757	4	54,55574977	-99,11149955	-83,61216741	4,936592618	160	0,031974836	log(T2_Mean) ~ genetic_clusters
0,12241199	0,0650533	1,029939819	2,134148863	0,0249301	11	-231,8508431	487,7016861	524,9000833	162,2987329	153	0,187484999	T2_Mean ~ island
0,127989601	0,05241537	1,036877443	1,693561328	0,067540845	14	-231,3280195	492,656039	539,1540354	161,2672248	150	0,188747889	T2_Mean ~ island+genetic_clusters
0,002286532	-0,0164206	1,073878507	0,12222783	0,946890342	4	-242,3705315	494,741063	510,2403951	184,5144076	160	0,195483374	T2_Mean ~ genetic_clusters

Porites sTL

r.squared	adj.r.squared	sigma	statistic	p.value	df	logLik	AIC	BIC	deviance	df.residual	error.rate	frml
**0,245278708**	**0,19179452**	**0,39062856**	**4,58600431**	**2,98463E-05**	**10**	**-60,42295518**	**142,8459104**	**174,9657006**	**19,37901533**	**127**	**0,040116012**	**log(sTL_Mean) ~ island**
0,259077024	0,1873748	0,391695192	3,613235561	0,000121033	13	-59,15900294	146,3180059	187,1977388	19,0247153	124	0,040225551	log(sTL_Mean) ~ island+genetic_clusters
0,054117079	0,03278137	0,427332008	2,536456098	0,059457359	4	-75,88821851	161,776437	176,3763416	24,28748177	133	0,043885311	log(sTL_Mean) ~ genetic_clusters
0,217285814	0,16181788	5,024730036	3,917322978	0,000203343	10	-410,3716278	842,7432556	874,8630458	3206,484815	127	0,516019947	sTL_Mean ~ island
0,223816321	0,14870177	5,063891619	2,97966668	0,001099035	13	-409,7977066	847,5954131	888,4751461	3179,731793	124	0,520041687	sTL_Mean ~ island+genetic_clusters
0,037532043	0,01582224	5,444779675	1,72880621	0,164137788	4	-424,5328499	859,0656998	873,6656044	3942,86822	133	0,559157388	sTL_Mean ~ genetic_clusters

Commonality analysis

Variable	Coefficient	Total %
Pocillopora hTL: log(hTL_Mean) ~ island + genetic_clusters (R2 adj = 0.13)		
Unique to island	0,1531	75,75
Unique to genetic_clusters	0,0612	30,29
Common to island, and genetic_clusters	-0,0122	-6,04
Total	0,2021	100
Pocillopora sTL: log(sTL_Mean) ~ island + genetic_clusters (R2 adj = 0.35)		
Unique to island	0,2278	54,13
Unique to genetic_clusters	0,057	13,55
Common to island, and genetic_clusters	0,136	32,31
Total	0,4208	100
Porites hTL: log(hTL_Mean) ~ island (R2 adj = 0.09)		
Porites sTL: log(sTL_Mean) ~ island (R2 adj = 0.19)		

Supplementary Table 5

Regression models between TL, island of sampling and genetic lineages for the 11 islands. r.squared: multiple R2; adj.r.squared: adjusted R2; sigma: Residual Standard Error (RSE); statistic: F-test value; p.value: p-value of the F-test; df: degree of freedom of the overall F-test; logLik: log-likelihood of the model; AIC: Akaike's Information Criterion; BIC: Bayesian Information Criterion; df.residual: Residual degrees of freedom; error.rate: RSE divided by the mean of TL; frml: formula of the model. The last sheet describes the commonality analysis (see Table 1 for details).

Variable	Type of environmental data
lat	Historical
lon	Historical
SST_min_DegC	Historical
SST_max_DegC	Historical
SST_mean_DegC	Historical
SST_std_DegC	Historical
seasonal_min_DegC	Historical
seasonal_max_DegC	Historical
seasonal_mean_DegC	Historical
seasonal_std_DegC	Historical
SST_anomaly_min_DegC	Historical
SST_anomaly_max_DegC	Historical
SST_anomaly_mean_DegC	Historical
SST_anomaly_std_DegC	Historical
SST_anomaly_freq_max_day	Historical
SST_anomaly_freq_mean_day	Historical
SST_anomaly_freq_std_day	Historical
TSA_heat_max_DegC	Historical
TSA_heat_mean_DegC	Historical
TSA_heat_std_DegC	Historical
TSA_heat_freq_max_day	Historical
TSA_heat_freq_sum_day	Historical
TSA_heat_freq_mean_day	Historical
TSA_heat_freq_std_day	Historical
TSA_DHW_max_DegC	Historical
TSA_DHW_sum_DegC	Historical
TSA_DHW_mean_DegC	Historical
TSA_DHW_std_DegC	Historical
TSA_DHW_freq_max_day	Historical
TSA_DHW_freq_sum_day	Historical
TSA_DHW_freq_mean_day	Historical
TSA_DHW_freq_std_day	Historical
TSA_DHW_minlenght_day	Historical
TSA_DHW_maxlenght_day	Historical
TSA_DHW_meanlenght_day	Historical
TSA_DHW_stdlenght_day	Historical
TSA_DHW_lastmax_DegC	Historical
TSA_DHW_lastsum_DegC	Historical
TSA_DHW_lastduration_day	Historical
TSA_DHW_minrecovery_day	Historical
TSA_DHW_maxrecovery_day	Historical
TSA_DHW_meanrecovery_day	Historical
TSA_DHW_lastrecovery_day	Historical
TSA_cold_min_DegC	Historical
TSA_cold_mean_DegC	Historical
TSA_cold_std_DegC	Historical
TSA_cold_freq_max_day	Historical
TSA_cold_freq_sum_day	Historical
TSA_cold_freq_mean_day	Historical
TSA_cold_freq_std_day	Historical
TSA_DCW_min_DegC	Historical
TSA_DCW_sum_DegC	Historical

TSA_DCW_mean_DegC	Historical
TSA_DCW_std_DegC	Historical
TSA_DCW_freq_max_DegC	Historical
TSA_DCW_freq_sum_day	Historical
TSA_DCW_freq_mean_day	Historical
TSA_DCW_freq_std_day	Historical
TSA_DCW_minlenght_day	Historical
TSA_DCW_maxlenght_day	Historical
TSA_DCW_meanlenght_day	Historical
TSA_DCW_stdlenght_day	Historical
TSA_DCW_lastmin_DegC	Historical
TSA_DCW_lastsum_DegC	Historical
TSA_DCW_lastduration_day	Historical
TSA_DCW_minrecovery_day	Historical
TSA_DCW_maxrecovery_day	Historical
TSA_DCW_meanrecovery_day	Historical
TSA_DCW_lastrecovery_day	Historical
Moon_light_lux	Contextual
barometer_hp	Contextual
airTemp	Contextual
dewPoint	Contextual
Course_Over_Ground	Contextual
Speed_over_Ground	Contextual
apparent_windspeed_bow	Contextual
apparent_winddir_bow	Contextual
true_wind_speed	Contextual
true_wind_dir	Contextual
gamma_acs_final	Contextual
fCDOM_ppb	Contextual
PAR	Contextual
ssh_copernicus	Contextual
u_copernicus	Contextual
v_copernicus	Contextual
current_speed_copernicus	Contextual
MLD_copernicus	Contextual
wavesignificantheight_copernicus	Contextual
windspeed_copernicus	Contextual
eastward_wind_copernicus	Contextual
orthward_wind_copernicus	Contextual
o2_copernicus	Contextual
nppv_copernicus	Contextual
phyc_copernicus	Contextual
spco2_copernicus	Contextual
mergedPOC	Contextual
SST	Contextual
SSS	Contextual
chl	Contextual
daily_min_zenith	Contextual
Aerosols_conc	Contextual
PAR_Sat	Contextual
PIC_Sat	Contextual
bb_GSM_Sat	Contextual
KD490_Sat	Contextual

NO2__mole_L_	Contextual
Proc_mL	Contextual
Syn_mL	Contextual
Pico_mL	Contextual
Nano_mL	Contextual
OrgNano_mL	Contextual
TotalEuks_mL	Contextual
CorrectedBact_mL	Contextual
corrected_AT	Contextual
corrected_CT	Contextual
NO3	Contextual
PO4	Contextual
SiOH	Contextual
pH	Contextual
MoonLength	Contextual
SunLength	Contextual

Supplementary Table 6: **List of the historical and contextual environmental data used for the sPLS analysis.**
