# Supplementary material for: Telomere DNA length regulation is influenced by seasonal temperature differences in short-lived but not in long-lived reef-building corals
Source: Nat Commun. 2023 Jun 1;14:3038. doi: 10.1038/s41467-023-38499-1 (PMC10235076; doi:10.1038/s41467-023-38499-1)
Supplement: Supplementary file 3 — Description of Additional Supplementary Files [file 41467_2023_38499_MOESM3_ESM.pdf]

Description of Additional Supplementary Files

File name: Supplementary Data 1

Description: Numerical values used for the sPLS of Figure 3 Values for each coral colony of hTL, sTL and historical/contextual environmental variables.

File name: Supplementary Data 2

Description: Values of R2 related to the variance partition analysis in Fig. 4a. The marginal R2 considers only the variance of the fixed effects, while the conditional R2 considers both the fixed and random effects (i.e., the total model). In addition, the variance attributable for each variable as well as the variance for the residual is given. The last column shows the variable attributed to each gene when the variance is >25%.

File name: Supplementary Data 3

Description: Functional analysis of hTL-corelated genes List of significant Biological Process pathways of the hTL-corelated genes whose expression variance is attributable to island, Symbiodiniaceae composition, host species and hTL (see Fig. 4a).
