# Supplementary material for: Telomere DNA length regulation is influenced by seasonal temperature differences in short-lived but not in long-lived reef-building corals
Source: Nat Commun. 2023 Jun 1;14:3038. doi: 10.1038/s41467-023-38499-1 (PMC10235076; doi:10.1038/s41467-023-38499-1)
Supplement: Supplementary file 7 — Reporting Summary [file 41467_2023_38499_MOESM7_ESM.pdf]

nature portfolio

Corresponding author(s): Eric Gilson & Alice Rouan

Last updated by author(s): 2023 03 15

Reporting Summary

Nature Portfolio wishes to improve the reproducibility of the work that we publish. This form provides structure for consistency and transparency in reporting. For further information on Nature Portfolio policies, see our Editorial Policies and the Editorial Policy Checklist.

Statistics

For all statistical analyses, confirm that the following items are present in the figure legend, table legend, main text, or Methods section.

n/a	Confirmed	
☐	☒	The exact sample size (*n*) for each experimental group/condition, given as a discrete number and unit of measurement
☐	☒	A statement on whether measurements were taken from distinct samples or whether the same sample was measured repeatedly
☐	☒	The statistical test(s) used AND whether they are one- or two-sided<br>*Only common tests should be described solely by name; describe more complex techniques in the Methods section.*
☐	☒	A description of all covariates tested
☐	☒	A description of any assumptions or corrections, such as tests of normality and adjustment for multiple comparisons
☐	☒	A full description of the statistical parameters including central tendency (e.g. means) or other basic estimates (e.g. regression coefficient) AND variation (e.g. standard deviation) or associated estimates of uncertainty (e.g. confidence intervals)
☐	☒	For null hypothesis testing, the test statistic (e.g. *F*, *t*, *r*) with confidence intervals, effect sizes, degrees of freedom and *P* value noted<br>*Give P values as exact values whenever suitable.*
☒	☐	For Bayesian analysis, information on the choice of priors and Markov chain Monte Carlo settings
☒	☐	For hierarchical and complex designs, identification of the appropriate level for tests and full reporting of outcomes
☐	☒	Estimates of effect sizes (e.g. Cohen's *d*, Pearson's *r*), indicating how they were calculated

Our web collection on statistics for biologists contains articles on many of the points above.

Software and code

Policy information about availability of computer code

Data collection	Telomere Restriction Fragment (TRF) images were generated with Typhoon (GE Healthcare) and signal was extracted using ImageQuantTL 7.0 (GE Healthcare).
Data analysis	R (v3.6.3) was used for statistical analysis and to generate plots (used packages are in brackets): data curation (readxl, stringi, stringr, reshape2, rowr, devtools and tidyverse) ,boxplots (ggplot2, RColorBrewer and plotly), correlograms (corrplot and GGally), functionnal analysis (topGO, Reactome and ggplot2), variance partition (variancePartition and ggplot2), sPLS (mixOmics), regression models and statistical analysis (stats, yhat and multcompView), maps (marmap and ggplot2). No custom code was generated.

For manuscripts utilizing custom algorithms or software that are central to the research but not yet described in published literature, software must be made available to editors and reviewers. We strongly encourage code deposition in a community repository (e.g. GitHub). See the Nature Portfolio guidelines for submitting code & software for further information.

Data

Policy information about availability of data

All manuscripts must include a data availability statement. This statement should provide the following information, where applicable:

- Accession codes, unique identifiers, or web links for publicly available datasets
- A description of any restrictions on data availability
- For clinical datasets or third party data, please ensure that the statement adheres to our policy

The Telomere DNA length (TL) data generated in this study have been deposited in the Ze-nodo database under accession code doi.org/10.5281/

zenodo.3999999ZZ. The other varia-bles used in this work are available as follows: environmental parameters: https://zenodo.org/record/6299409#.YkRwrTdBzjA; RNAseq: doi.org/10.5281/zenodo.7398767; symbiodinaceae (ITS2) community: https://doi.org/10.5281/zenodo.4061796; bacterial (16S rRNA) communi-ty: https://doi.org/10.5281/zenodo.4073268; colony size: doi.org/10.1101/2022.10.13.512013; species delimitation: doi.org/10.1101/2022.10.13.512013 and https://doi.org/10.1101/2022.10.21.513203. Source data are provided with this paper.

Human research participants

Policy information about studies involving human research participants and Sex and Gender in Research.

Reporting on sex and gender	N/A
Population characteristics	N/A
Recruitment	N/A
Ethics oversight	N/A

Note that full information on the approval of the study protocol must also be provided in the manuscript.

Field-specific reporting

Please select the one below that is the best fit for your research. If you are not sure, read the appropriate sections before making your selection.

☐ Life sciences ☐ Behavioural & social sciences ☒ Ecological, evolutionary & environmental sciences

For a reference copy of the document with all sections, see nature.com/documents/nr-reporting-summary-flat.pdf

Ecological, evolutionary & environmental sciences study design

All studies must disclose on these points even when the disclosure is negative.

Study description	We measured telomeric DNA length (TL) of coral samples with the gold standard procedure named TRF (for Terminal Restriction Fragment), allowing to calculate the coral host TL (TTAGGG)n (named hTL) and symbiont TL (named sTL). In total, we measured TL of 851 colonies collected from 99 reef sites around 32 islands across the Pacific Ocean (443 colonies of Pocillopora spp. and 408 colonies of Porites spp.). (see Supplementary Figure 4).
Research sample	DNA extracted from coral fragments of Pocillopora spp. (five species including P. meandrina) and of Porites spp. (three species including P. Lobata).
Sampling strategy	10 coral replicates for each genus sampled at three differentes reef sites around the 32 islands. The colonies were chosen based on their ressemblance to P.Meandrina and P. lobata. Colonies of healthy apparence were preferentially sampled. Samples were flash-frozen in liquid nitrogen on board and kept stored at -20°C before DNA extraction and TL analysis. Full description is provided with manuscript presenting the full protocol and methodology of all Tara pacific sampling (Lombard et al, Scientific Data, in press, https://www.biorxiv.org/content/10.1101/2022.05.25.493210v1).
Data collection	Data concerning sample collection were recorded by the divers/scientists in the field using various log sheets which are publicly available at : https://store.pangea.de/Projects/TARA-PACIFIC/Logsheets/
Timing and spatial scale	Sampling of coral colonies occurred over a two year period (2016-2028) as part of the Tara Pacific Expedition. Colonies were sampled from 99 reef sites around 32 islands spanning nearly 17 000 km overwater distance across the Pacific Ocean.
Data exclusions	Samples with significant DNA degradation were excluded from the analysis. Moreover, TRF Southern blots without signal or with a poor quality were removed from the analysis.
Reproducibility	The mean TLs estimated from independent TRF experiments are highly correlated (Pearson's test, R =0.7233599;p<2.2e-16;n=226 for hTL, and R = 0,8953802; p<2.2e-16;n=117). See the Method section and Supplementary Figure 3.
Randomization	N/A
Blinding	Two blinded observers assessed signal quality of the TRF Southern blots by reporting lanes without signal or with a poor quality one; these were removed from the analysis.

Did the study involve field work? ☒ Yes ☐ No

Field work, collection and transport

Field conditions	Relevant environmental parameters for each island/site are summarized in Supplementary Table 7 of the manuscript. These include data for both at the time of sampling as well as relevant historical conditions (from Lombard et al, Scientific Data, in press, https://www.biorxiv.org/content/10.1101/2022.05.25.493210v1 and https://zenodo.org/record/6299409#.YkRwrTdBzjA).
Location	The 32 islands that were sampled are: Islas de las Perlas, Coiba (two samplings: one in 2016, one in 2018), Malpelo, Rapa Nui, Ducie, Gambier, Moorea, Aitutaki, Niue, Upolu, Futuna, Tuvalu, Abaiang, Chuuk, Guam, Ogasawara, Okinawa, Fiji, Heron, Chesterfield, New Caledonia, Solomon, Milney Bayk, Kimbe Bay, Palau South Isands, Palau , Hong Kong, Taiwan, Hawaii, Baja California, Clipperton.
Access & import/export	Sampling permit for PANAMA under the reference 'SE/AP-18-16' delivered by the Direccion de Areas Protegidas y Vida Silvestre - LIC. Samuel Valdez Diaz Director - Ministerio de Ambiente – Republica de Panama on the 13/06/2016; Sampling permit for PANAMA under the reference '2016-0701-2019-2' delivered by the Smithsonian Tropical Research InstituteInstituto Smithsonian de Investigaciones Tropicales - STRI Animal Care and Use Committee (ACUC) on the 28/06/2016; Sampling permit for PANAMA under the reference '2016-0701-2019-2-A1' delivered by the Smithsonian Tropical Research InstituteInstituto Smithsonian de Investigaciones Tropicales - STRI Animal Care and Use Committee (ACUC) on the 21/06/2018; Sampling permit for COLOMBIA under the reference 'N°009' delivered by the MINISTERIO DE AMBIENTE Y DESARROLLO SOSTENIBLEPARQUES NACIONALES NATURALES DE COLOMBIA on the 04/03/2016; Sampling permit for CHILE under the reference '13270/24/457/Vrs' delivered by the Servicio Hidrografico y Oceanografico de la Armada de Chile (SHOA) – Patricio Carrasco Hellwig Contraalmirante Director on the 29/08/2016; Sampling permit for UNITED-KINGDOM (PITCAIRN ISLANDS) under the reference 'N/A' delivered by the Government of Pitcairn islands /Environmental, Conservation & Natural Resources Division Manager // Christian Michele on the 25/02/2016; Sampling permit for COOK under the reference '11-16' delivered by the Foundation for National Research – Cook Island Research Committee – Office of the Prime Minister – Elizabeth Wright-Koteka (Chairperson) on the 12/09/2016; Sampling permit for NIUE under the reference '34/16' delivered by the Government of Niue – Office for External Affairs on the 17/11/2016; Sampling permit for SAMOA under the reference 'Memorandum of Agreement' delivered by the THE GOVERNMENT OF THE INDEPENDENT STATE OF SAMOA acting by and through the Ministry of Natural Resources and Environment on the 29/11/2016; Sampling permit for WALLIS AND FUTUNA under the reference 'Arrêté n°2016-527' delivered by the Le Préfet, Administrateur supérieur des îles Wallis et Futuna on the 24/11/2016; Sampling permit for TUVALU under the reference 'MFAT : 449/16' delivered by the Government of Tuvalu – Ministry of Foreign Affairs on the 19/12/2016; Sampling permit for KIRIBATI under the reference '015/16' delivered by the Environment and Conservation Division – Republic of Kiribati on the 24/11/2016; Sampling permit for MICRONESIA under the reference 'Letter' delivered by the Deputy Assistant Secretary – Marine Resources Unit – Department of Resources and Development – Federated States of Micronesia on the 05/04/2017; Sampling permit for GUAM under the reference 'U2021-023' delivered by the Marine. Scientific Research CoordinatorOffice of Ocean and Polar Affairs – United States Department of StateBureau of Oceans and InternationalEnvironmental and Scientific Affairs on the 27/10/2021; Sampling permit for AMERICAN SAMOA under the reference 'U2021-022' delivered by the Marine Scientific Research CoordinatorOffice of Ocean and Polar Affairs – United States Department of StateBureau of Oceans and InternationalEnvironmental and Scientific Affairs on the 27/10/2021; Sampling permit for FIJI under the reference '456/2017' delivered by the Ministry of Foreign Affairs – Republic of Fiji on the 11/06/2017; Sampling permit for AUSTRALIA under the reference 'G17/39873.1' delivered by the Great Barrier Reef Marine Park Authority and Department of Foreign Affairs and Trade on the 30/08/2017; Sampling permit for NEW-CALEDONIA (SOUTH PROVINCIA) under the reference 'Arrêté n°2720-2017/ARR/DENV modifiant l'arrêté 1515-2017/ARR/DENV du 04 août 2017' delivered by the Président de l'Assemblée de la Province Sud de la Nouvelle-Calédonie on the 06/09/2017; Sampling permit for NEW-CALEDONIA (CHESTERFIELD) under the reference 'Arrêté n° 2017-2069/GNC' delivered by the Haut-Commissariat de la République en Nouvelle-Calédonie – Gouvernement de Nouvelle-Calédonie – République Française on the 29/08/2017; Sampling permit for SOLOMON ISLANDS under the reference 'Form 01 (?)' delivered by the Solomon Islands Maritime Safety Administration on the 20/09/2017; Sampling permit for PAPUA NEW-GUINEA under the reference '907/2017 (diplomatic clearance n°0232)' delivered by the Department of Foreign Affairs and Trade of the Independent State of Papua New Guinea on the 27/10/2017; Sampling permit for PALAU under the reference 'RE-18-04' delivered by the Ministry of Natural Resources, Environment and Tourism – Republic of Palau on the 21/12/2017; Sampling permit for CHINA (HONG-KONG) under the reference 'CMO-N00811' delivered by the Marine Department, Hong-Kong, China on the 15/03/2018; Sampling permit for USA (HAWAII) under the reference 'U2018-010' delivered by the United States Department of StateBureau of Oceans and InternationalEnvironmental and Scientific Affairs on the 06/06/2018; Sampling permit for MEXICO under the reference 'PPF/DGOPA-291/17' delivered by the Secrataria de Agricultura, Ganaderia, Desarrollo rural, pesca y alimentacion – Comision Nacional de Acuacultura y Pesca – Direccion General de Ordenamiento Pesquero y Acuicola – Estados Unidos Mexicanos on the 28/08/2018; Sampling permit for CLIPPERTON under the reference 'HC/1195/CAB' delivered by the Haut-Commissariat de la République Polynésie Française on the 13/06/2018; Sampling permits for JAPAN under the reference '28-50' delivered by the Prefecture of Tokyo on the 01/23/2017 for sampling in the Tokyo Prefecture (Ogasawara Island), '28-74' delivered by the Prefecture of Okinawa on the 04/14/2017 for sampling in the Okinawa Prefecture (Sesoko Island) and 'N/A' delivered by the Ministry of Agriculture, Forestry and Fisheries on the 01/10/2017 for sampling in Japanese EEZ. Sampling Permit for Taiwan has been obtained but need to be translated to be displayed here. Export/import CITES permits have been accorded for those different countries.
Disturbance	sampling was restricted to the minimal amount of material and did not involved environmentally destructive methods.

Reporting for specific materials, systems and methods

We require information from authors about some types of materials, experimental systems and methods used in many studies. Here, indicate whether each material, system or method listed is relevant to your study. If you are not sure if a list item applies to your research, read the appropriate section before selecting a response.

Materials & experimental systems

n/a	Involved in the study	
☒	☐	Antibodies
☒	☐	Eukaryotic cell lines
☒	☐	Palaeontology and archaeology
☐	☒	Animals and other organisms
☒	☐	Clinical data
☒	☐	Dual use research of concern

Methods

n/a	Involved in the study	
☒	☐	ChIP-seq
☒	☐	Flow cytometry
☒	☐	MRI-based neuroimaging

Animals and other research organisms

Policy information about studies involving animals; ARRIVE guidelines recommended for reporting animal research, and Sex and Gender in Research

Laboratory animals	This study does n,ot involve laboratory animals.
Wild animals	Fragments of Pocillopora and Porites colonies (ca. 2 inches in length) were clipped from living corals in the field, transported onboard of the tara vessel where they were flashed frozen and kept at -20°C until they were sent to France (usually less than two months).
Reporting on sex	N/A
Field-collected samples	The frozen coral samples were transported at -20°C until the IRCAN laboratory in Nice (France) were high molecular weight DNA was extracted.
Ethics oversight	No ethical approvance or guidance was necessary since organismms sampled were invertebrates and are not concerned. Any environmental or ethical guidance proposed by the different sampling permits were followed and sampling was restriced to the minimal amount of material and did not involved environmentaly destructive methods.

Note that full information on the approval of the study protocol must also be provided in the manuscript.
